# Antioxidant Bioactivity of Particalized Eggshell Membrane (PEM) Targeting Potential Functional Food Applications

**DOI:** 10.3390/foods15213906

**Published:** 2026-10-30

**Authors:** Tamer A. E. Ahmed, Manar Younes, Riadh Hammami, Maxwell T. Hincke

**Affiliations:** 1 Department of Cellular and Molecular Medicine, Faculty of Medicine, University of Ottawa, Ottawa, ON K1H 8M5, Canada; tahmed@uottawa.ca (T.A.E.A.); myoun141@uottawa.ca (M.Y.); 2 School of Nutrition Sciences, Faculty of Health Sciences, University of Ottawa, Ottawa, ON K1H 8M5, Canada; riadh.hammami@uottawa.ca; 3 Department of Innovation in Medical Education, Faculty of Medicine, University of Ottawa, Ottawa, ON K1H 8M5, Canada

**Keywords:** eggshell membrane, bioactives, antioxidant, functional foods, alkaline hydrolysis, nutraceuticals

## Abstract

Eggshell membrane (ESM) is a food-processing poultry by-product containing over 500 proteins and a valuable source of bioactive peptides that remain minimally exploited for functional foods and nutraceuticals. The current study developed a distinct valorization and fractionation strategy using clean, size-selected particalized ESM (PEM) as a defined feedstock to generate peptide-containing fractions with improved antioxidant and potential intestinal barrier-supportive activities. PEM batches (<53 μm) were subjected to alkaline hydrolysis using potassium hydroxide (KOH), followed by neutralization, fractionation, simulated gastrointestinal digestion, and desalting. Bioactivities of different fractions were evaluated using Trolox equivalent antioxidant capacity (TEAC), nitric oxide (NO) production in LPS-stimulated RAW264.7 macrophages, and transepithelial electrical resistance (TEER) in differentiated Caco-2 intestinal epithelial monolayers. KOH hydrolyzed the crosslinked ESM protein meshwork, with increased recovery of soluble proteins and peptides, achieving ~78% solubilization. The soluble PEM hydrolysate fraction (PS) exhibited the greatest reduction in LPS-induced NO production, whereas its intestinal digestion product (PSI) showed 65-fold greater TEAC compared to unprocessed PEM. Several PEM-derived fractions reduced LPS-induced NO production and improved TEER responses. These findings demonstrate that alkaline hydrolysis and gastrointestinal digestion markedly alter the bioactivity of ESM-derived fractions, supporting further investigation of these fractions as a multifunctional ingredient for potential food and nutraceutical applications.

## 1. Introduction

Oxidative stress is characterized by the accumulation of reactive oxygen species (ROS) and plays a key role in the development of most chronic conditions [1]. An imbalance between the generated ROS and cellular antioxidant mechanisms leads to oxidative stress that disrupts cellular homeostasis through deoxyribonucleic acid (DNA) damage, protein oxidation, and lipid peroxidation [2,3]. Excessive ROS production has been linked to chronic inflammation and contributes to the pathogenesis of inflammatory, neurodegenerative, cardiovascular, and metabolic diseases, as well as neoplastic disorders [1,2,3,4]. Oxidative stress contributes indirectly to the continuously increasing healthcare expenditure and socioeconomic demands associated with these chronic conditions [5]. The gastrointestinal tract (GIT) represents a key player in controlling systemic oxidative homeostasis, as gut dysbiosis, altered intestinal barrier function, and chronic mucosal inflammation can promote excessive ROS generation, contributing to systemic inflammatory and metabolic disorders [6,7]. Accordingly, antioxidant approaches targeting gastrointestinal health and redox homeostasis have attracted interest as potential strategies to prevent disease and promote health [2,7].

Food-derived functional ingredients, particularly those produced through the valorization of agricultural and food-processing by-products, represent promising sources of bioactive compounds with antioxidant, anti-inflammatory, and health-promoting properties [8,9]. Various extraction and bioprocessing strategies have been developed to recover proteins, peptides, and other bioactive components from these underutilized sources [9]. This valorization approach aligns with the circular economy principles by reducing waste accumulation and generating high-value functional ingredients for food and nutraceutical applications [10]. In this context, eggshell (ES) and its associated membrane (ESM), a major byproduct of the egg processing industry, represent promising but underexploited biomaterials [11].

Globally, approximately 100 million tons of table eggs are produced annually, generating substantial quantities of ES and ESM waste [12]. ESM constitutes around 1% of the egg weight; its proteinaceous meshwork contains over 500 proteins with diverse functionalities [11]. In addition to its role in protecting the developing embryo and providing structural support for ES assembly, ESM possesses structural, physicochemical, and biological properties suitable for a vast array of biomedical applications [13,14]. However, despite its unique fibrous nature and protein composition, ESM has a highly crosslinked protein network and poor aqueous solubility, diminishing the release of functional bioactives [13,14,15,16]. Although powdered ESM offers improved surface area, its antioxidant, anti-inflammatory, and antimicrobial capacities remain limited, as most of the bioactive proteins remain sequestered within the insoluble matrix [15,16,17,18]. In contrast, soluble ESM proteins and ESM hydrolysates containing low-molecular-weight peptides possess significantly enhanced bioavailability and antioxidant properties; however, their production can require complex processing protocols [15,17,19,20,21,22]. In addition to its demonstrated antioxidant activity, ESM possess various bioactivities, including antimicrobial properties, along with its capacity to modulate immune responses [23].

Despite these promising properties, further exploration is needed to assess how processing, fractionation, and simulated gastrointestinal digestion influence the composition and bioactivities of ESM-derived fractions. We hypothesize that a valorization strategy of ESM waste via optimized solubilization strategies can generate peptide-containing ingredients with improved antioxidant capacity. Building on our previous study of Jet-O-Mized ESM, which highlighted the feasibility of generating hydrolyzed ESM with gut-health-promoting bioactivities [23], where bulk ESM material supplied by Burnbrae Farms (BBF) was further processed using Jet milling to produce a finer feedstock, the present study investigates how a distinct processing and fractionation approach using clean, size-selected PEM as a prepared in-house feedstock influences the distribution and bioactivity of ESM-derived fractions. A scalable upstream process for producing intact ESM with very low residual calcium and controlled microbiological quality has been developed and is the subject of a patent filing [24], with the resulting ESM feedstock intended for subsequent particle-size reduction, fractionation, and hydrolysis to generate bioactive fractions. The current study evaluates various chemical solubilization strategies, compares acid- and alkaline-mediated processing, and assesses the resulting whole, soluble, and insoluble fractions following alkaline hydrolysis before and after simulated gastrointestinal digestion, with particular emphasis on their antioxidant potential. Accordingly, the objectives of this study were to (i) investigate various chemical solubilization strategies for PEM, (ii) optimize processing conditions to improve protein solubilization while maintaining microbiological quality, and (iii) characterize the resulting whole, soluble, and insoluble fractions and their changes following simulated gastrointestinal digestion to determine their antioxidant capacity, effects on lipopolysaccharide (LPS)-induced NO production in 264.7 murine macrophage cell line (RAW264.7 macrophages), and effects on transepithelial electrical resistance (TEER) in human colorectal adenocarcinoma cell line (Caco-2 cells).

## 2. Materials and Methods

### 2.1. Materials

The cell lines used in the study included RAW264.7 macrophages (American Type Culture Collection, ATCC^®^ TIB-71™, Lot#70012232, Manassas, VA, USA) and Caco-2 cells (ATCC, HTB-37, Manassas, VA, USA). Unless stated otherwise, all additional chemicals and supplies were procured from Fisher Scientific, Ottawa, ON, Canada, or MilliporeSigma, Oakville, ON, Canada.

### 2.2. Experimental Design

**Figure 1:**
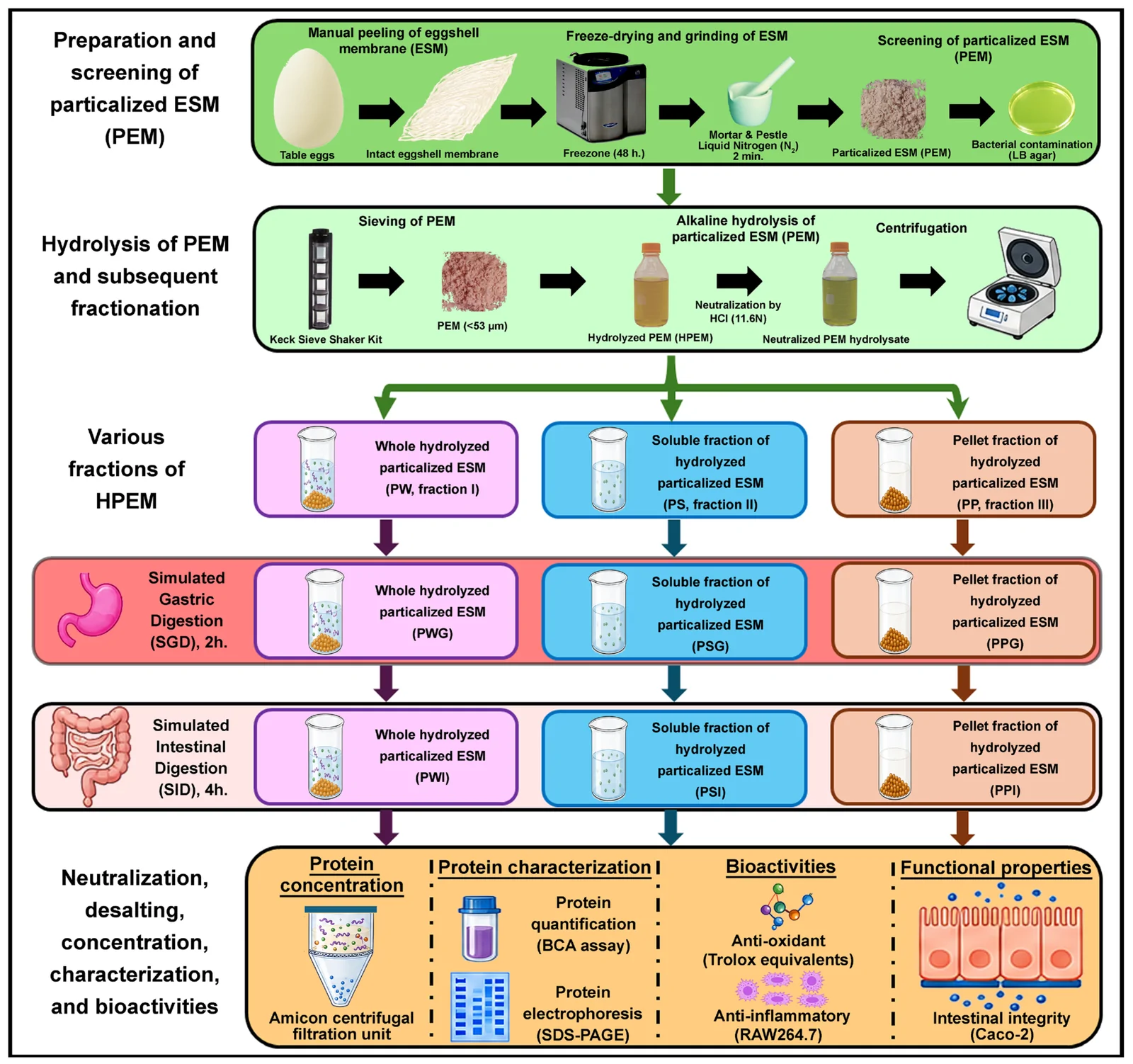
Schematic representation of PEM processing, followed by purification and characterization of PEM-derived peptides and digestion products and their subsequent in vitro assessment.

The study involved the preparation and evaluation of PEM fractions following chemical processing and simulated gastrointestinal digestion (SGID). The prepared fractions were assessed for antioxidant capacity and their effects on LPS-induced nitric oxide (NO) production in RAW264.7 macrophages and intestinal barrier integrity in Caco-2 monolayers. The experimental workflow and relationships among the preparation procedures and subsequent analyses are illustrated in Figure 1. The preparation of the PEM biomaterial and the individual fractions, together with the corresponding analytical procedures, is described in the following subsections.

### 2.3. Particalized Eggshell Membrane (PEM) Biomaterial

Particalized eggshell membrane (PEM) was prepared as previously described [18]. Briefly, ESM was manually peeled from intact graded white-shell eggs supplied by Burnbrae Farms (BBF). The manually isolated ESM was freeze-dried for 48 h (Freezone, Labconco) and cryo-ground for 2 min in the presence of liquid nitrogen to produce particalized eggshell membrane (PEM) powder. PEM (<53 μm) batches show no detectable bacterial growth using agar plate assays. Fractionation of PEM was achieved by sieving using a Keck Sieve Shaker Kit (Cole-Parmer, Montreal, QC, Canada) to generate different fractions, including PEM with sizes < 53, 53–104, 104–381, and 381–508 μm. PEM with size < 53 μm was used for further processing.

### 2.4. Preliminary Acid and Alkaline Hydrolysis Screening

A systematic preliminary hydrolysis screening was first carried out using five acids (acetic, hydrochloric, nitric, phosphoric, and sulfuric acids), selected to represent both organic and inorganic acids with different acid strengths, dissociation characteristics, and chemical properties. This selection enabled a preliminary comparison of the effects of different acidic environments on PEM solubilization. The acids were evaluated across a range of concentrations (0.125 N, 0.375 N, and 1.25 N) and temperatures (ambient, 50, and 70 °C), with all conditions maintained under static incubation to assess their potential for PEM solubilization, with solubility monitored over 4 days at five time points (days 0, 1, 2, 3, and 4). Change in turbidity, by measuring optical density at 600 nm (OD_600_), was used as an indicator of solubilization rather than as a direct measurement of protein solubilization. Alkaline hydrolysis was evaluated using only two bases, potassium hydroxide (KOH) and sodium hydroxide (NaOH). Preliminary KOH hydrolysis trials therefore investigated key processing parameters, including temperature, hydrolysis time, PEM and KOH concentrations, and stirring intensity, for the production of hydrolyzed PEM (HPEM) containing soluble proteins and peptides [23,25]. After completion of the hydrolysis, the reaction was terminated by neutralization with 11.6 M hydrochloric acid, with the hydrolysate adjusted to pH 7.0. Thereafter, the entire hydrolysate (PW, fraction I) was centrifuged to separate two additional fractions, including the soluble fraction (PS, containing soluble proteins and peptides; fraction II) from the insoluble pellet (PP, fraction III). The neutralization endpoint and centrifugation parameters used for the collection of different HPEM fractions are also part of the protected processing procedure. All fractions were stored at −20 °C for subsequent analysis and processing.

### 2.5. Simulated Gastrointestinal Digestion of PEM Hydrolysate Fractions

The whole (PW), soluble (PS), and pellet (PP) fractions of the PEM hydrolysate were separately subjected to simulated gastrointestinal digestion (SGID) as previously described [26]. The SGID procedure was based on the standardized in vitro digestion approach described by Minekus et al. (2014) [26], with modifications to the digestion conditions as described below. The digestion consisted of sequential simulated gastric digestion (SGD) and simulated intestinal digestion (SID) phases. This sequential approach was used to simulate the exposure of PEM hydrolysate fractions to the gastric and intestinal environments. PEM is predominantly proteinaceous, with carbohydrates and lipids representing only minor constituents [11,12]. Accordingly, both digestion phases were included to evaluate the effects of sequential gastric and intestinal proteolysis on PEM-derived fractions. Herein, the term “digestion products” refers to digestion-derived products released from PEM hydrolysate fractions following SGID, including proteins, protein fragments, peptides, and other soluble digestion-derived compounds.

In the gastric phase, each PEM hydrolysate fraction was incubated with simulated gastric fluid (SGF) and pepsin at 37 °C for 2 h, with the pH adjusted to 3.0 using concentrated hydrochloric acid (HCl). The gastric digestion was conducted under controlled temperature and acidic conditions to mimic the gastric environment and facilitate pepsin-mediated digestion of the PEM-derived material. Following gastric digestion, samples were neutralized and stored at −20 °C for subsequent analysis. The resulting digestion products were designated PWG, PSG, and PPG for the whole, soluble, and pellet fractions, respectively.

For the intestinal phase, the gastric digests were subsequently incubated with simulated intestinal fluid (SIF), bile salts, and pancreatin at 37 °C for an additional 4 h, with the pH adjusted to 7.0 using NaOH. This intestinal phase was designed to simulate the subsequent exposure of the gastric digestion products to near-neutral intestinal conditions and pancreatic digestive enzymes. The inclusion of bile salts in the intestinal phase was intended to approximate the intestinal digestion environment rather than to specifically investigate lipid digestion. The resulting intestinal digestion products were designated PWI, PSI, and PPI, respectively, and were desalted and stored at −20 °C until further processing and analysis. The detailed compositions of the simulated gastric and intestinal digestion media and the digestion components used in the present study are provided in Supplementary Table S1.

### 2.6. Desalting and Concentration of PEM Hydrolysate Fractions and Digests

Insoluble protein and particulate materials were removed before desalting by centrifugation at 14,000 rpm for 30 min at 4 °C (Refrigerated benchtop centrifuge 5430 R, Eppendorf Canada Limited, Mississauga, ON, Canada) to prevent blockage and subsequent reduced filtration efficiency of the centrifugal filtration units. No desalting was performed before or during the simulated gastrointestinal digestion stages. Consequently, residual salts generated during hydrolysate neutralization, including KCl formed through the reaction of KOH with HCl, may have been present during digestion. The potential contribution of these residual salts to ionic strength and digestive enzyme activity was not independently evaluated. The clear PEM hydrolysate fractions and their gastrointestinal digestion products were subjected to desalting and concentration before downstream characterization and bioactivity assessment. Centrifugal filter units (Millipore™ Amicon™ Ultra-15, MilliporeSigma, Oakville, ON, Canada; 3 kDa molecular weight cutoff) were used to remove residual salts and concentrate the soluble protein and peptide fractions. Because the 3-kDa MWCO may lead to loss of very-low-molecular-weight peptides and other small digestion products, the recovered material was considered a protein- and peptide-containing fraction. Samples were processed according to the manufacturer’s instructions (MilliporeSigma, Oakville, ON, Canada), and the concentrated fractions were collected and stored at −20 °C until further analysis. The overall processing scheme, including PEM hydrolysis, fractionation, simulated gastrointestinal digestion, and desalting/concentration, is summarized in Figure 1.

### 2.7. Protein Quantification and SDS-PAGE Profiling of PEM Hydrolysate Fractions and Digestion Products

Protein content and molecular-weight distribution of PEM hydrolysate fractions and their simulated gastrointestinal digestion products were evaluated. Soluble protein concentration was determined using the bicinchoninic acid (BCA) assay (Pierce BCA Protein Assay Kit, Thermo Fisher Scientific, Rockford, IL, USA), following the manufacturer’s instructions, with absorbance measured at 562 nm [18]. The soluble protein concentrations measured by BCA were used to calculate protein solubilization percentage (%) relative to the total protein weight of the starting PEM material according to the following equation:

**Protein solubilization percentage (%) = (RSP/IPW) × 100**

RSP: Recovered soluble protein as determined by BCA.

IPW: Initial protein weight (g) of the starting insoluble PEM.

Sodium dodecyl sulfate–polyacrylamide gel electrophoresis (SDS-PAGE) was used to qualitatively characterize the apparent molecular-weight distribution of soluble protein constituents using precast polyacrylamide gels (NuPAGE 4–12% Bis-Tris Gel, Cat.no. NP0321Box, Fisher Scientific) and Coomassie Brilliant Blue staining [27]. SDS-PAGE profiles were utilized to assess the shift in the apparent molecular-weight distribution following hydrolysis and subsequent SGID, rather than to provide precise molecular-weight identification or quantify protein solubilization.

### 2.8. Assessment of Bioactivities of the Developed PEM Formulations

The whole hydrolysate (PW) and pellet fraction (PP) were subjected to SGID to liberate soluble digestion products for subsequent bioactivity assessment. Following digestion, each preparation was centrifuged to separate the insoluble material from the soluble digestion products. Only the resulting soluble supernatants were used for the subsequent bioactivity and functional assessments. Serial dilutions of each desalted and concentrated supernatant were prepared based on the soluble protein and peptide concentrations and subsequently evaluated for bioactivity.

#### 2.8.1. Total Antioxidant Capacity

The Trolox equivalent antioxidant capacity (TEAC) of various PEM-derived soluble fractions was determined using the Total Antioxidant Power Kit (Oxford Biomedical Research, Rochester Hills, MI, USA; Product No. TA02), according to the manufacturer’s instructions. The assay is based on a copper-reducing antioxidant capacity principle, similar to the CUPRAC method [28], in which antioxidants reduce Cu^2+^ to Cu^+^, followed by formation of a colored Cu^+^–neocuproine complex measured at 450 nm. This assay was selected to provide a consistent measure of total reducing antioxidant capacity for direct comparison among the different PEM-derived fractions and their simulated gastrointestinal digestion products. Briefly, the samples and Trolox standards were diluted in the supplied dilution buffer, and antioxidant capacity was determined from the net absorbance measured at 450 nm after correction using the reference absorbance at 600 nm [18,23]. The dilution buffer was used as the reagent blank. The antioxidant capacity was expressed as Trolox equivalents.

#### 2.8.2. Effect on LPS-Induced Nitric Oxide Production in RAW264.7 Macrophages

The potential anti-inflammatory activity of the PEM-derived soluble fractions and their gastrointestinal digestion products was evaluated using lipopolysaccharide (LPS)-stimulated RAW 264.7 macrophages by measuring nitric oxide (NO) accumulation in the culture medium as an indicator of the inflammatory response. RAW 264.7 macrophages were cultured in Dulbecco’s modified Eagle medium (DMEM) supplemented with 10% heat-inactivated fetal bovine serum, penicillin–streptomycin, and glutamine at 37 °C in a humidified atmosphere containing 5% CO_2_, and subcultured at ~70% confluency using 0.25% trypsin-EDTA (Ethylenediaminetetraacetic acid) (Gibco, Grand Island, NY, USA). Thereafter, cells were treated with PEM-derived test fractions at a concentration of 0.5 mg/mL in the presence of 1 μg/mL LPS for 24 h. Following treatment, culture media were collected and centrifuged, and NO accumulation was quantified using the Griess Reagent System (Promega, Madison, WI, USA), according to the manufacturer’s instructions [25]. Cells treated with LPS in the absence of PEM-derived material served as a positive inflammatory control (LC), whereas cells treated with the test material without LPS served as the negative control. NO production was calculated using a nitrite standard curve. In a different experimental setting, cell viability was assessed using the alamarBlue^®^ assay to confirm that the tested concentrations did not adversely affect cell viability, which in turn can alter the cellular production of NO.

### 2.9. Effect of PEM-Derived Digestion Products on Transepithelial Electrical Resistance of Caco-2 Monolayers

The effects of PEM-derived fractions and their gastrointestinal digestion products on electrical resistance of Caco-2 cell monolayers were evaluated by measuring changes in transepithelial electrical resistance (TEER), which was used as an indicator of epithelial barrier integrity under the experimental conditions. Caco-2 cells were cultured in DMEM supplemented with 10% heat-inactivated fetal bovine serum, penicillin–streptomycin, and glutamine at 37 °C in a humidified atmosphere containing 5% CO_2_. Cells were seeded onto 0.4 μm-pore-size cell culture inserts (ThinCerts^TM^, Cat. No. 665640, culture surface 1.131 cm^2^) and maintained until a stable TEER of approximately 300 Ω·cm^2^ was achieved after 21 days, indicating formation of a differentiated intestinal epithelial barrier and strong tight junctions [29,30]. TEER was measured using a Millicell ERS-2 (MilliporeSigma), and the area-normalized TEER was calculated according to the equation: TEER (Ω·cm^2^) = [R (Cells) − R (Blank)] × insert surface area (113.1 mm^2^), where R (Cells) is the measured resistance of the cell-containing insert, and R (Blank) is the resistance of a cell-free insert. Different concentrations of PEM-derived test fractions were prepared using culture media (0.5 mL) and added to the apical compartment of the Caco-2 monolayers. TEER was measured before exposure and after 24 h to assess changes in monolayer integrity. Non-treated Caco-2 cells served as the negative control (NC), while ionomycin was used as a positive control (IC) for tight-junction disruption [30].

### 2.10. Statistical Analysis

Statistical analyses were carried out using GraphPad Prism (version 10). Statistical differences among the treatment and corresponding control (CTRL) groups were assessed using one-way analysis of variance (ANOVA), followed by Dunnett’s multiple-comparisons test. PEM fractions were derived from a common preparation obtained by pooling three separately extracted samples. Data are presented as the mean ± standard deviation derived from three independent biological experiments conducted in technical triplicate for each assay. Technical triplicates were averaged before statistical analysis, with independent experiments (*n* = 3) serving as the statistical units for various in vitro assays. For all analyses, a *p*-value < 0.05 was considered statistically significant. Where applicable, statistical significance is indicated in the figures using the corresponding *p*-value thresholds. Graphs and figures were generated and assembled using GraphPad Prism and Adobe Photoshop CS (v 8.0). GraphPad Prism was used for data visualization and statistical analysis, while Adobe Photoshop CS (v 8.0) and BioRender were used, where appropriate, for figure assembly, schematic representation, and illustration of proposed mechanisms and experimental concepts. Figure formatting and graphical presentation were standardized, where appropriate, to facilitate comparison of the experimental findings across the different assays.

## 3. Results

### 3.1. Screening of Acids and Bases for PEM Hydrolysis

Particalized eggshell membrane (PEM) used in this study was obtained by manual peeling of ESM from the egg interior, followed by grinding and sieving to collect PEM with particle size < 53 µm. PEM was subsequently treated with various acids or bases to evaluate their ability to solubilize the crosslinked ESM protein fibrous network and release soluble proteins and peptides with potential bioactivity. Initial screening of various acids, including acetic, hydrochloric, nitric, phosphoric, and sulfuric acids, at three different concentrations (0.125, 0.375, and 1.25 N) was carried out to evaluate PEM solubilization under static conditions at 50 °C. A gradual decrease in optical density at 600 nm (OD_600_) occurred with PEM solubilization and was therefore monitored as an indicator for extent of hydrolysis. Most acid treatments, covering a range of acid strengths and concentrations, did not lead to a consistent or substantial reduction in OD_600_ indicative of enhanced PEM solubilization (Figure S1). Nitric acid (1.25 N) was an exception, as it elicited a marked decrease in OD_600_ after several days of incubation. However, this solubilization required relatively high concentration and extended time (3–4 days), which is not compatible with the environmentally friendly and scalable processing approach envisioned for future process development. Likewise, incubation at much lower or higher temperatures (ambient and 70 °C) did not result in noticeable hydrolysis. Therefore, acid-mediated hydrolysis was not pursued further in this study.

In contrast, alkaline hydrolysis was evaluated using two bases, potassium hydroxide (KOH) and sodium hydroxide (NaOH). KOH was subsequently selected for further optimization, as it exhibited a solubilization response comparable to that of NaOH (Figure S2) under preliminary screening conditions. In addition, KOH had not previously been investigated for ESM solubilization based on our literature search. Preliminary KOH hydrolysis trials evaluated key processing parameters, including temperature, hydrolysis time, PEM and KOH concentrations, and stirring intensity, for the production of hydrolyzed PEM (HPEM) containing soluble proteins and peptides. The KOH-mediated hydrolysis conducted under increased agitation and elevated temperature conditions led to improved solubilization, as indicated by decreased OD_600_ values under the conditions evaluated. OD_600_ was used as a screening measure of solubilization, while soluble protein recovery was additionally assessed using the BCA assay.

### 3.2. Protein Quantification and SDS-PAGE Profiling of PEM Hydrolysate Fractions and Digestion Products

**Figure 2:**
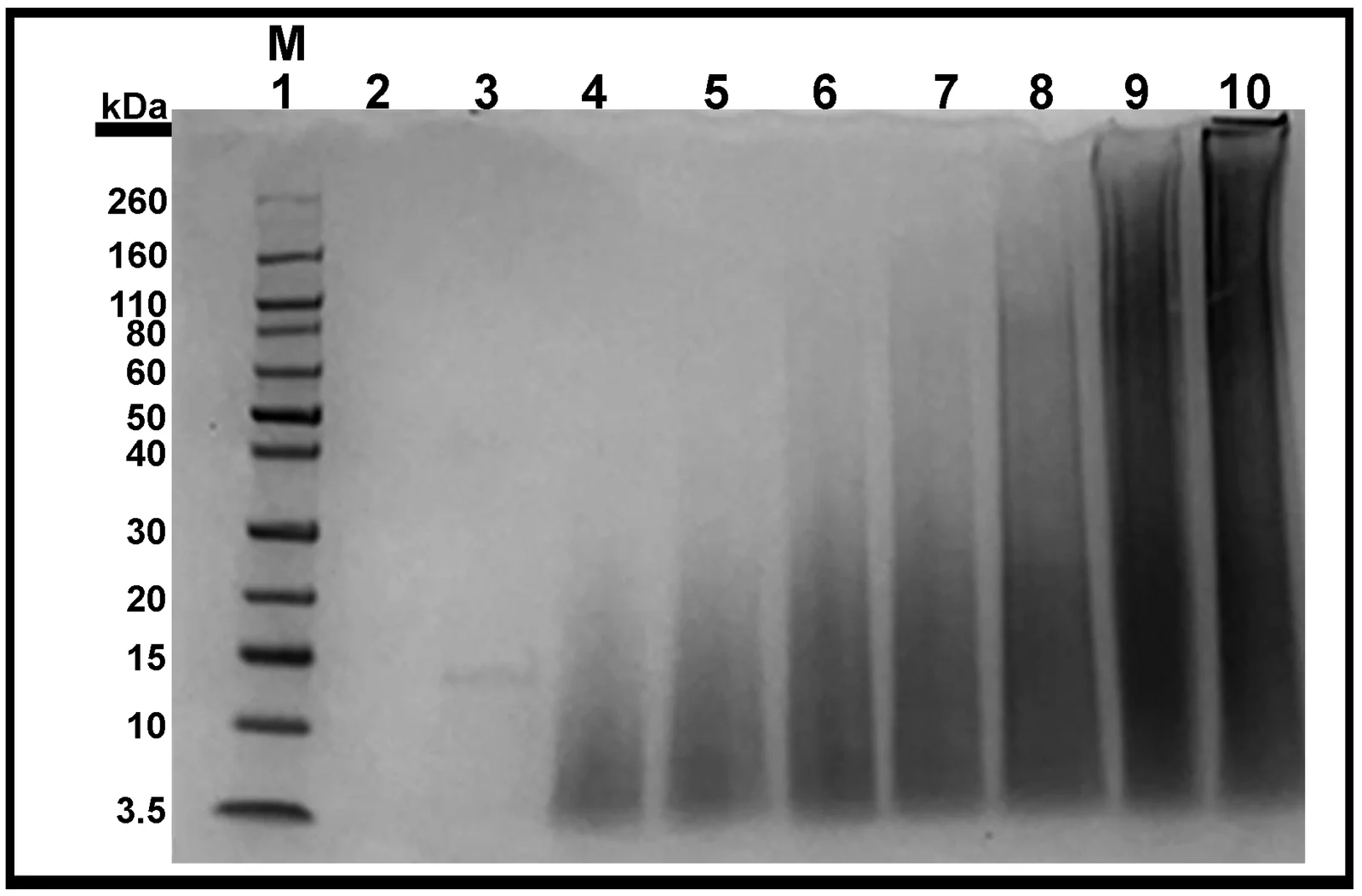
SDS-PAGE profiling of time-dependent KOH-mediated hydrolysis of PEM. Time-dependent hydrolysis of PEM using KOH. Lane 1, prestained molecular weight marker (M); lane 2, empty lane; lanes 3–10, KOH-mediated PEM hydrolysis at 0, 8, 11, 13, 15, 17, 21, and 24 h, respectively, showing the developing changes in the protein profile with increasing hydrolysis time.

**Figure 3:**
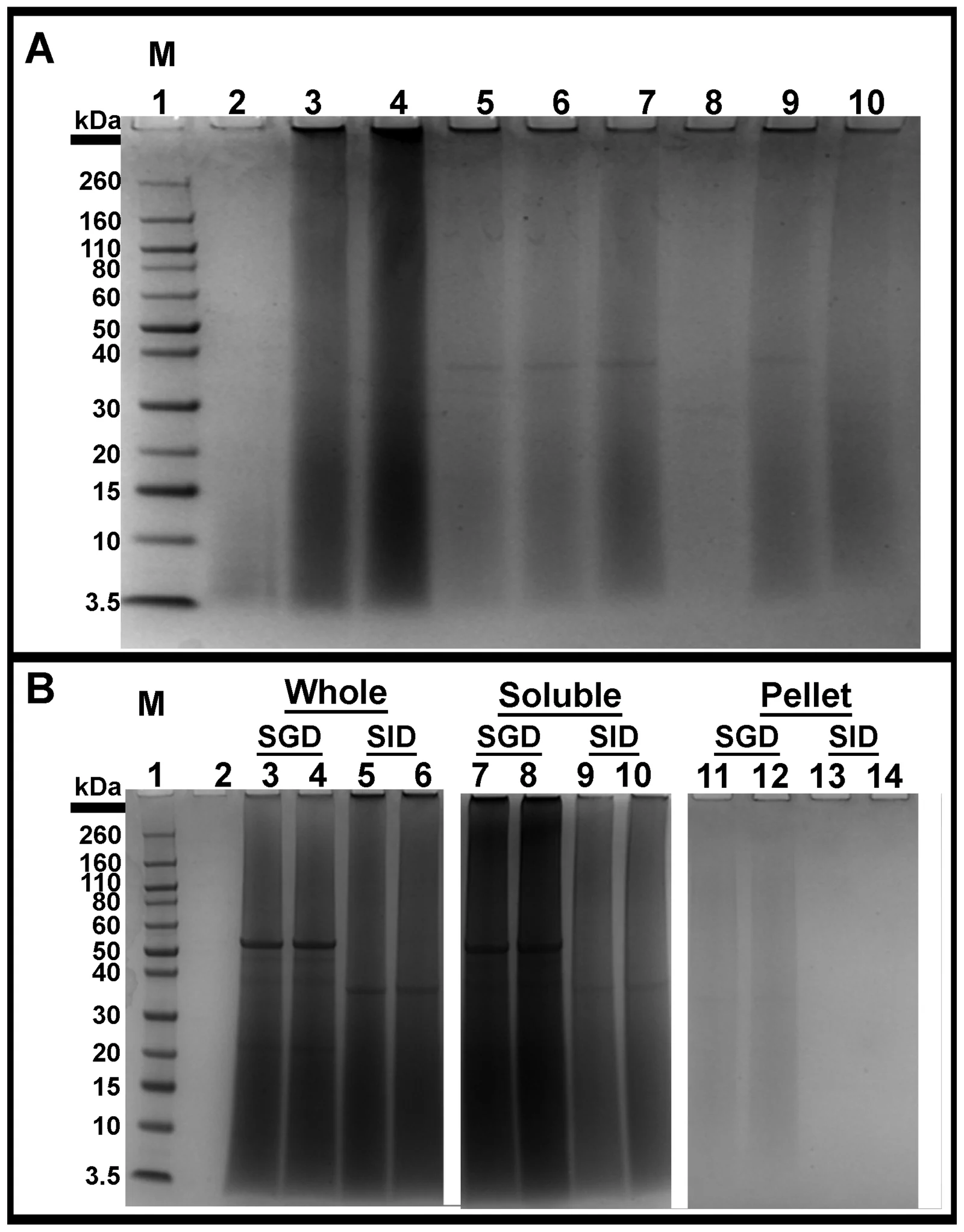
SDS-PAGE profiling of KOH-hydrolyzed PEM and its simulated gastrointestinal digestion products. (**A**) SDS-PAGE profiles of soluble KOH-hydrolyzed PEM before and after simulated gastrointestinal digestion and subsequent desalting/concentration. Lane 1, prestained molecular weight marker (M); lane 2, PEM hydrolysate at 0 h; lane 3, PEM hydrolysate after 18 h of KOH hydrolysis; lane 4, 18 h hydrolysate after desalting and concentration using an Amicon centrifugal ultrafiltration unit; lane 5, hydrolysate before simulated gastric digestion (SGD); lane 6, hydrolysate after 2 h of SGD; lane 7, hydrolysate after 2 h of SGD followed by desalting and concentration; lane 8, hydrolysate before simulated intestinal digestion (SID); lane 9, hydrolysate after 4 h of SID; and lane 10, hydrolysate after 4 h of SID followed by desalting and concentration. All samples in lanes 2–10 were derived from the soluble fraction of KOH-hydrolyzed PEM. (**B**) SDS-PAGE profiles of whole, soluble, and pellet PEM fractions following simulated gastrointestinal digestion. Lane 1, prestained molecular weight marker (M); lane 2, empty lane; lanes 3 and 4, whole hydrolysate following 2 h of SGD (two independent batches); lanes 5 and 6, whole hydrolysate following 4 h of SID (two independent batches); lanes 7 and 8, soluble PEM fraction following 2 h of SGD (two independent batches); lanes 9 and 10, soluble PEM fraction following 4 h of SID (two independent batches); lanes 11 and 12, pellet PEM fraction following 2 h of SGD (two independent batches); and lanes 13 and 14, pellet PEM fraction following 4 h of SID (two independent batches). SGD, simulated gastric digestion; SID, simulated intestinal digestion; PEM, particalized eggshell membrane; kDa: kilodalton.

The soluble protein concentrations of the PEM-derived fractions and their gastrointestinal digestion products were quantified using the BCA assay. The KOH-mediated hydrolysis process generated soluble proteins and peptides, with a BCA-based quantification indicating approximately 78% protein solubilization, while SDS-PAGE qualitatively highlighted the substantial shift in the apparent molecular-weight distribution. SDS-PAGE was used thereafter to assess changes in the protein profiles of PEM during KOH-mediated hydrolysis and subsequent simulated gastrointestinal digestion (Figure 2 and Figure 3). Progressive changes in the protein profile were observed with increasing KOH hydrolysis time (Figure 2). At shorter hydrolysis times, the staining was localized mainly in the lower-molecular-weight region, below approximately 20 kDa, indicating the presence of soluble low-molecular-weight proteins and peptide fragments. With extended hydrolysis time, the staining progressively expanded across a broader apparent molecular-weight range, with smearing observed from approximately 3.5 to 260 kDa.

The soluble KOH-hydrolyzed PEM fraction was subsequently evaluated before and after simulated gastric digestion (SGD) and simulated intestinal digestion (SID) (Figure 3A). The KOH hydrolysate at 18 h exhibited a distinctly altered electrophoretic profile compared to the non-hydrolyzed material at 0 h, with staining distributed across a broad molecular-weight range and most intense below 30 kDa. Following desalting and concentration, which involved a 3-kDa molecular-weight cutoff, the protein profile was observed with improved visualization of the soluble components. Therefore, very-low-molecular-weight species below the 3-kDa cutoff were not retained for subsequent analysis. Subsequent SGD and SID produced further changes in the electrophoretic patterns, although substantial low-molecular-weight material remained detectable after both digestion stages. Desalting and concentration after digestion similarly enhanced the visualization of the retained soluble protein/peptide fractions.

To further examine the electrophoretic profile of PEM-derived digestion products following gastrointestinal digestion, whole, soluble, and pellet fractions were analyzed by SDS-PAGE (Figure 3B). The whole hydrolysate displayed a visible protein smear extending from approximately 50 kDa into the lower-molecular-weight region, with broadly similar profiles observed between the two independent batches. The soluble fractions exhibited an intense staining pattern across a broad molecular-weight range (3.5–260 kDa) following both SGD and SID, with more intense staining toward the lower-molecular-weight region. In contrast, the pellet fractions showed substantially weaker staining, indicating that most of the SDS-PAGE-soluble peptides/proteins were associated with the soluble fraction following simulated gastrointestinal digestion. In general, the SDS-PAGE profiles demonstrate that KOH hydrolysis substantially altered the molecular-weight distribution of PEM-derived hydrolysate and that the resulting soluble material remained detectable following simulated gastric and intestinal digestion.

### 3.3. Assessment of Bioactivities of the PEM-Derived Digestion Products

Serial dilutions of PEM-derived digestion products were prepared based on the concentration of soluble proteins and peptides obtained from each treatment after desalting, and their activities were measured.

#### 3.3.1. Total Antioxidant Capacity

**Figure 4:**
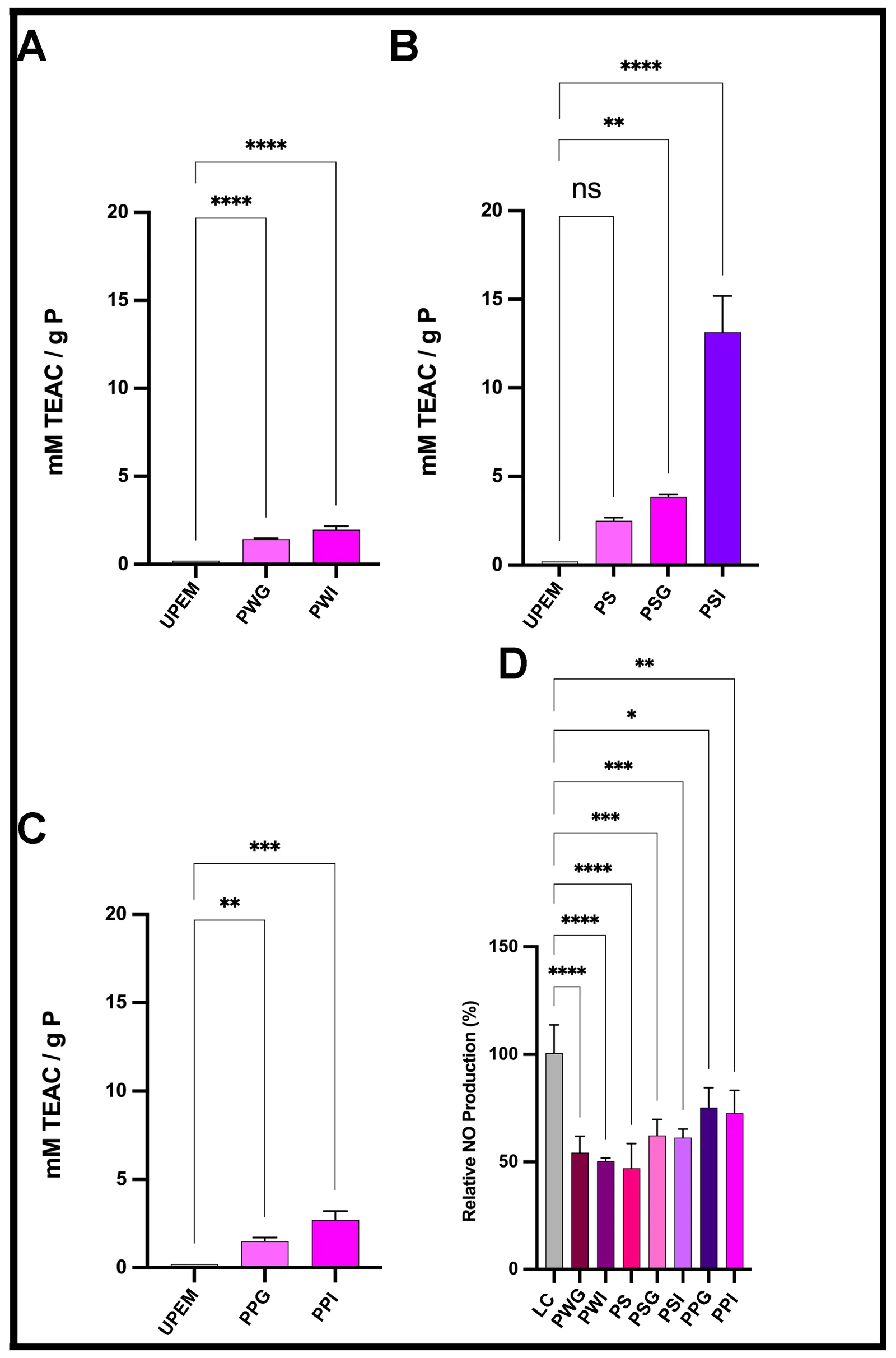
Antioxidant and anti-inflammatory activities of particalized eggshell membrane (PEM) fractions and simulated gastrointestinal digestion products. (**A**) Trolox equivalent antioxidant capacity (TEAC) of whole PEM digestion products obtained after simulated gastric (PWG) and intestinal (PWI) digestion. (**B**) TEAC of the soluble PEM fraction before (PS) and after simulated gastric (PSG) and intestinal (PSI) digestion. (**C**) TEAC of the pellet PEM fractions after simulated gastric (PPG) and intestinal (PPI) digestion. TEAC values of the different PEM fractions and digestion products were compared to unprocessed eggshell membrane (UPEM). (**D**) Relative nitric oxide (NO) production by LPS-stimulated RAW 264.7 macrophages treated with different PEM fractions and digestion products (0.5 mg/mL). NO accumulation was compared with that of LPS-induced cells (LC). Data are presented as mean ± SD. Statistical significance is indicated as *p* < 0.05 (*), *p* < 0.01 (**), *p* < 0.001 (***), and *p* < 0.0001 (****); ns, not significant.

The total antioxidant capacity of the PEM-derived fractions and their gastrointestinal digestion products was evaluated using the Trolox equivalent antioxidant capacity (TEAC) assay and expressed as mM Trolox equivalents (mM TEAC). The antioxidant capacity varied considerably among the PW, PS, and PP fractions and their respective gastrointestinal digestion products (Figure 4 and Table 1). For the whole fraction (PW), PWG and PWI exhibited significantly higher TEAC values of 1.4 ± 0.0 and 2.0 ± 0.2 mM (*p* < 0.0001), respectively, as compared to control unprocessed PEM (UPEM, 0.2 ± 0.0 mM). The soluble fraction (PS) showed a greater variation in antioxidant capacity. PS exhibited a TEAC value of 2.5 ± 0.2 mM, which increased markedly following gastric digestion (PSG) to 3.8 ± 0.2 (*p* < 0.01) and intestinal digestion (PSI) to 13.1 ± 2.1 mM (*p* < 0.0001), with PSI exhibiting the highest TEAC value among all tested fractions and digestion products. Similarly, for the pellet fraction (PP), PPG and PPI exhibited TEAC values of 1.5 ± 0.2 (*p* < 0.01) and 2.7 ± 0.5 mM (*p* < 0.001), respectively, showing significantly higher antioxidant capacity than the control UPEM. The dramatic increase observed for PSI suggests that the antioxidant capacity of the soluble fraction was altered following intestinal digestion. The TEAC assay reflects the overall antioxidant capacity of the samples under the assay conditions and does not identify individual antioxidant compounds or specific mechanisms responsible for the observed activity.

#### 3.3.2. Effects on LPS-Induced NO Production in RAW264.7 Macrophages

The effects of PEM-derived digestion products on LPS-induced NO production were assessed using RAW 264.7 macrophages, with relative nitric oxide (NO) accumulation used as an indicator of the inflammatory response. The effects varied among the whole, soluble, and pellet fractions and their corresponding digestion products (Figure 4 and Table 1). For the whole fraction (I), relative NO production was 54 ± 8% for PWG and 50 ± 2% for PWI, which were significantly lower (*p* < 0.0001) than the lipopolysaccharide control (LC, 101 ± 13), indicating a reduction in LPS-induced NO production following both gastric and intestinal digestion. Similarly, for the soluble fraction (II), PS, PSG, and PSI displayed significantly reduced NO levels of 47 ± 12% (*p* < 0.0001), 62 ± 8% (*p* < 0.001), and 61 ± 4% (*p* < 0.001), respectively, as compared to the LPS control. Thus, the gastrointestinal digestion products showed modest inhibition of LPS-induced NO production, whereas the undigested soluble fraction (PS) exhibited the greatest inhibition of NO production among all PEM-derived digestion products. Regarding the pellet fraction (III), PPG and PPI produced relative NO levels of 75 ± 9 (*p* < 0.05) and 73 ± 11% (*p* < 0.01), respectively, and were significantly lower than the positive control (LC). These findings demonstrate fraction- and digestion-dependent differences in the modulation of LPS-stimulated NO production. The whole-fraction digestion products (PWG and PWI), along with the soluble fraction (PS), showed the greatest inhibition of relative NO production, whereas the soluble fraction digestion products (PSG and PSI) exhibited a moderate inhibition of NO accumulation. In contrast, the pellet fraction digestion products (PPG and PPI) displayed the least inhibition of NO production among all active PEM-derived products, with relative NO levels of 75% and 73%, respectively (Table 1).

### 3.4. Effect of PEM-Derived Digestion Products on Caco-2 Monolayer TEER

**Figure 5:**
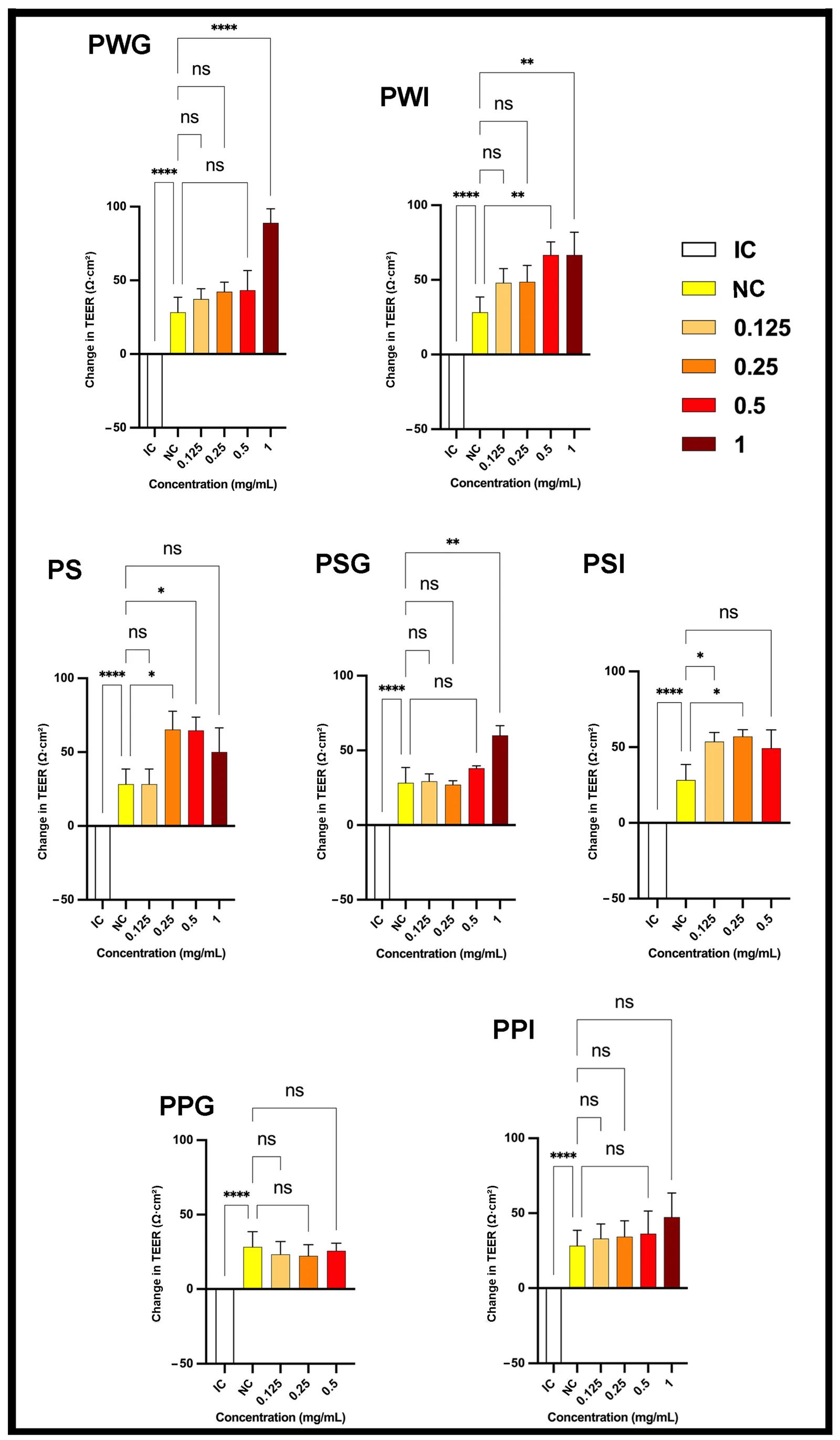
Effects of PEM-derived digestion products on transepithelial electrical resistance (ΔTEER) of Caco-2 cell monolayers. Caco-2 monolayers were treated with PEM-derived digestion products for 24 h, and changes in TEER (ΔTEER; Ω·cm^2^) were determined relative to the initial TEER measurement. PWG and PWI represent the whole fraction of KOH-hydrolyzed PEM following simulated gastric and intestinal digestion, respectively. PS, PSG, and PSI represent the soluble fraction before digestion and following simulated gastric and intestinal digestion, respectively. PPG and PPI represent the pellet fraction following simulated gastric and intestinal digestion, respectively. IC: ionomycin control (1 µM). Data are presented as mean ± SD. Statistical significance is indicated as *p* < 0.05 (*), *p* < 0.01 (**), and *p* < 0.0001 (****); ns, not significant. Brackets indicate statistical comparisons performed between the indicated groups.

Cell viability remained within 86–110% following exposure to all tested PEM-derived fractions and digestion product concentrations for both RAW264.7 macrophages and Caco-2 cells, with no statistically significant differences compared to the corresponding non-treated controls. The effects of PEM-derived digestion products on the integrity of differentiated Caco-2 cell monolayers were assessed 24 h post-treatment at concentrations ranging from 0.125 to 1 mg/mL, where applicable. Caco-2 monolayers were differentiated for 21 days, and experiments were initiated at a baseline TEER of approximately 300 Ω·cm^2^. Changes in TEER (ΔTEER) relative to the corresponding initial measurements at time zero were used as an indicator of changes in epithelial barrier integrity. The internal control (IC, ionomycin, 1 μM) showed a decrease in TEER over the 24-h period, confirming disruption of epithelial barrier integrity, whereas treatment with the PEM-derived digestion products generally resulted in higher TEER relative to the IC. The non-treated monolayer cells (0 mg/mL) were utilized as a baseline control for evaluating whether the treatments maintained or improved barrier integrity, while comparison with the IC provided a reference for barrier disruption. The effects varied among the whole, soluble, and pellet fractions and their corresponding digestion products (Table 2, Figure 5). Both whole hydrolysate digestion products (PWG and PWI), especially at the highest concentration (1 mg/mL), exhibited positive changes in TEER as compared to the non-treated monolayer cells (0 mg/mL). PWG displayed a dose-dependent pattern, with ΔTEER increasing progressively from approximately 28 ± 10 Ω·cm^2^ at 0 mg/mL to approximately 89 ± 10 Ω·cm^2^ at 1 mg/mL, with the greatest increase observed at the highest concentration. Similarly, PWI showed increasing TEER responses with increasing concentration, reaching approximately 67 ± 15 Ω·cm^2^ at 1 mg/mL. PWG at 1 mg/mL (*p* < 0.0001) and PWI at 0.5 and 1 mg/mL (*p* < 0.01) showed significant increases in ΔTEER relative to the non-treated control, whereas some pairwise comparisons among treatment concentrations were not statistically significant. For the soluble fraction, PS showed positive changes in TEER at all concentrations tested, with the greatest increases observed at 0.25 (65 ± 12 Ω·cm^2^) and 0.5 mg/mL (65 ± 9 Ω·cm^2^) (*p* < 0.05), compared to the non-treated monolayer cells. PSG displayed a nonsignificant TEER increase at concentrations of 0.125–0.5 mg/mL, followed by a significant increase at 1 mg/mL (approximately 60 ± 7 Ω·cm^2^), as compared to non-treated cells. In contrast, PSI significantly increased TEER at lower concentrations (54 ± 6 Ω·cm^2^ for 0.125 mg/mL and 57 ± 5 Ω·cm^2^ for 0.25 mg/mL; *p* < 0.05) as compared to the non-treated cells, although the response decreased slightly at 0.5 mg/mL. Finally, the pellet-derived digestion products displayed moderate and more consistent changes in TEER for different concentrations that were not significantly different from the non-treated cells, with no clear concentration-dependent pattern. In general, the PEM-derived digestion products generally produced higher Caco-2 monolayer TEER compared with the internal control, although the magnitude and concentration-response pattern varied according to the fraction and gastrointestinal digestion stage (Figure 5 and Table 2).

## 4. Discussion

The current study assessed the optimized processing of particalized eggshell membrane (PEM) with substantially reduced calcium content in order to test the hypothesis that solubilizing the highly crosslinked ESM protein network would liberate proteins—and peptides—containing fractions with improved antioxidant capacity. Although NaOH is more commonly reported for alkaline processing of proteinaceous biomass, KOH-based hydrolysis of PEM remains comparatively underexplored.

### 4.1. Processing and Generation of Soluble Proteinaceous Material

The highly crosslinked protein meshwork of ESM can hinder the accessibility of its constituent proteins and peptides. Previous studies have shown that chemical treatment and enzymatic hydrolysis, along with thermal and mechanical processing, can promote ESM solubilization and generate fractions with enhanced bioactivity [17,19,21,23,31,32,33,34,35,36,37].

In the current study, alkaline hydrolysis of PEM similarly promoted the recovery of soluble proteinaceous material, supporting the notion that disruption of the crosslinked ESM meshwork can improve accessibility of proteinaceous components. KOH was selected following preliminary screening, as it provided PEM solubilization comparable to NaOH under the conditions evaluated and, based on our literature search, had not previously been investigated for ESM solubilization. The process described herein successfully generated soluble proteins and peptide fractions, with a BCA-based quantification indicating approximately 78% protein solubilization, while SDS-PAGE qualitatively demonstrated a substantial shift in the apparent molecular-weight distribution. This protein solubilization was higher than the values reported in our previous study using a different ESM processing approach [23]. In addition, the present study used a defined, size-selected PEM feedstock rather than the bulk ESM subjected to Jet-O-Mizer processing in our previous study [25] and therefore represents a different processing and fractionation strategy. The resulting PW, PS, and PP fractions differed in their compositional characteristics and biological activities, including antioxidant capacity, suppression of LPS-induced NO production, and effects on Caco-2 monolayer TEER. These findings are consistent with previous evidence that ESM bioactivity is strongly affected by the processing strategy rather than being an intrinsic property of the intact membrane [11,13,18,23,26]. The present study extends previous work [23] by assessing the bioactivity of distinct PEM-derived fractions before and after simulated gastrointestinal digestion, rather than focusing solely on the activity of a bulk hydrolysate. A broader comparison of ESM processing strategies and reported bioactivities [17,19,20,21,22,23,25,31,32,33,34,35,36,37,38,39,40] is provided in Supplementary Table S2.

### 4.2. Antioxidant Capacity

The antioxidant capacity of ESM has been investigated using various chemical, enzymatic, thermal, fermentation, and structural modification strategies. As summarized in Table S2, these approaches can unleash different antioxidant properties and generate novel value-added bioactive fractions. Previous studies have reported enhanced ESM antioxidant capacity following enzymatic hydrolysis [19,21,31,32,33,36], thermal processing [17,34], subcritical water extraction [35], fermentation [37], and polyphenol incorporation [38], highlighting the influence of processing strategies on ESM-derived bioactivity. Our previous study further demonstrated that a scalable combination of ultrafine jet milling and KOH hydrolysis markedly enhanced the antioxidant capacity of ESM-derived materials, achieving a 15-fold increase compared with non-hydrolyzed ESM. The resulting soluble fraction also demonstrated antibacterial and anti-inflammatory activities, supporting ESM-derived peptides as multifunctional ingredients for gut health applications [23,25]. The findings of these studies highlighted that ESM antioxidant capacity is largely affected by processing and peptide accessibility.

Consistent with the literature-based evidence, our findings showed substantial improvements in the total antioxidant capacity of PEM and its gastrointestinal digestion products, indicating that antioxidant capacity was strongly influenced by fractionation and subsequent digestion. The gastrointestinal digestion products of the PW, PS, and PP fractions showed improved overall antioxidant capacity, with PSI digestion products exceeding 65-fold TEAC compared to unprocessed PEM. This improved antioxidant capacity may be due to the generation or exposure of novel peptide species following gastrointestinal digestion, although the present TEAC reflects the overall antioxidant capacity under the experimental conditions and does not identify individual antioxidant constituents or the underlying mechanism modulating the observed activity. Identification and characterization of the peptides responsible for this activity will require future peptide purification, sequencing, and structure–activity analyses.

### 4.3. Effects on LPS-Induced NO Production

ESM is a multifunctional biomaterial with anti-inflammatory activity that varies according to its physical format (powdered, solubilized, and digested) [11,13,18,23,26]. Unprocessed and digested natural eggshell membrane (NEM^®^) extracts have been reported to modulate cytokine production in an in vitro cell model [39]. More recent studies demonstrated that ESM hydrolysates and molecular-weight fractions also reduced NO production in LPS/ interferon-gamma (IFN-γ)-stimulated RAW264.7 macrophages, with activity depending on the evaluated fraction [22]. In our previous study, JEM-derived soluble and digested preparations reduced LPS-induced NO production by up to 80% in RAW264.7 macrophages while maintaining Caco-2 barrier compatibility; proteomics identified YBX1 and YWHAE among potential immunomodulatory effectors [23]. Various studies have also reported inhibition of inflammatory mediators, including tumor necrosis factor-alpha (TNF-α), interleukin (IL)-8, IL-1β, IL-6, nuclear factor kappa B (NF-κB), and inducible nitric oxide synthase (iNOS), in cellular models [19,40,41,42,43,44,45,46]. In vivo studies further support the anti-inflammatory activity of ESM-derived materials in models of intestinal inflammation and osteoarthritis [41,42,47,48,49,50].

Consistent with these findings, our results further demonstrate that the capacity of ESM-derived materials to suppress LPS-induced NO production in RAW264.7 macrophages depends on the nature of the fraction. The soluble PEM fraction (PS) exhibited the highest inhibition of LPS-induced NO production, indicating that solubilization increases the bioavailability of components capable of modulating macrophage inflammatory responses. However, gastrointestinal digestion did not further enhance this activity. In fact, PS retained superior reduction of LPS-induced NO production than its gastric and intestinal digestion products, suggesting that further digestion alters or degrades components responsible for the observed NO-suppressive effect. The relative viability of both RAW264.7 macrophages and Caco-2 cells remained between 86% and 110% across the tested concentrations, with no statistically significant differences compared with the respective negative controls. These findings indicate that the observed reduction in LPS-induced NO production was not associated with cytotoxicity under the experimental conditions used. These findings are consistent with previous work indicating that the concentration of PEM-derived materials and their gastrointestinal digestion products at concentrations up to 1 mg/mL was compatible with macrophage viability [18,23]. Previous studies likewise indicate that ESM hydrolysate activity depends on the processing strategy, molecular-weight distribution, the assessed dose, and the used cellular model [22,40].

### 4.4. Effect on Intestinal Integrity

Multiple studies have confirmed the potential of ESM and ESM-derived bioactives to support intestinal epithelial function [19,41,42]. However, these earlier studies did not directly measure transepithelial electrical resistance (TEER), paracellular permeability, or tight-junction integrity. Rønning et al. (2024) demonstrated that micronized ESM powder was not transported across differentiated Caco-2 monolayers, while the ESM peptide fraction was transported [47]. Interestingly, the hydrolysate fractions did not significantly alter expression of claudin-1 (*CLDN1*) or tight junction protein 1 (*TJP1*) in TNF-α-stimulated Caco-2 cells, indicating that peptide transport does not necessarily require concomitant changes in tight-junction protein expression [47]. Our recent report on jet-milled ESM (JEM) demonstrated that soluble JEM-derived fractions and gastrointestinal digestion products increased TEER in differentiated Caco-2 monolayers without increasing FITC-dextran permeability [23].

Consistent with these findings, the present study showed that PEM-derived digestion products generally improved Caco-2 monolayer integrity relative to the positive control, although the magnitude of the response varied according to fraction and digestion status. Whole hydrolysate and soluble fractions, including their digestion products, displayed greater effects on TEER, while pellet-derived digestion products produced moderate effects without a significant concentration-dependent pattern. These results indicate that ESM solubilization may enhance the ability of PEM-derived fractions to increase Caco-2 monolayer TEER under the conditions tested. The present TEER findings provide evidence of altered barrier resistance rather than direct evidence of tight-junction remodeling, particularly because TEER alone does not establish changes in specific junctional proteins or paracellular permeability.

In vivo evidence also supports a potential role for ESM in intestinal homeostasis, although most studies have examined indirect indicators rather than direct measurements of epithelial permeability or resistance. Dietary ESM improved microbial balance, microbial diversity, beneficial bacterial abundance, and cecal butyrate while reducing inflammatory responses in IL-10 knockout mice [48,49]. ESM supplementation in an azoxymethane/dextran sodium sulfate (AOM/DSS)-induced model also increased colonic *Cldn1* expression without significantly altering occludin (*Ocln*) or *Tjp1* expression [50]. These findings support potential intestinal benefits but do not establish generalized restoration of tight-junction integrity. This in vivo evidence supports ESM-mediated enhancement in epithelial restitution and intestinal homeostasis, while direct measurements of intestinal permeability and barrier resistance remain scarce.

### 4.5. Data-Driven Summary of Antioxidant, LPS-Induced NO Production, and Caco-2 TEER Responses

Oxidative stress contributes to epithelial damage, inflammation, and barrier dysfunction [51,52,53,54], with recent evidence linking intestinal barrier impairment with systemic inflammatory and oxidative responses [54,55]. Herein, the antioxidant effect, reduction in LPS-induced NO production, and increases in Caco-2 TEER observed for PEM-derived digestion products may represent interconnected mechanisms contributing to intestinal and potentially systemic homeostasis [51,52,53,54,55]. However, these relationships remain speculative and require validation in in vivo models.

**Figure 6:**
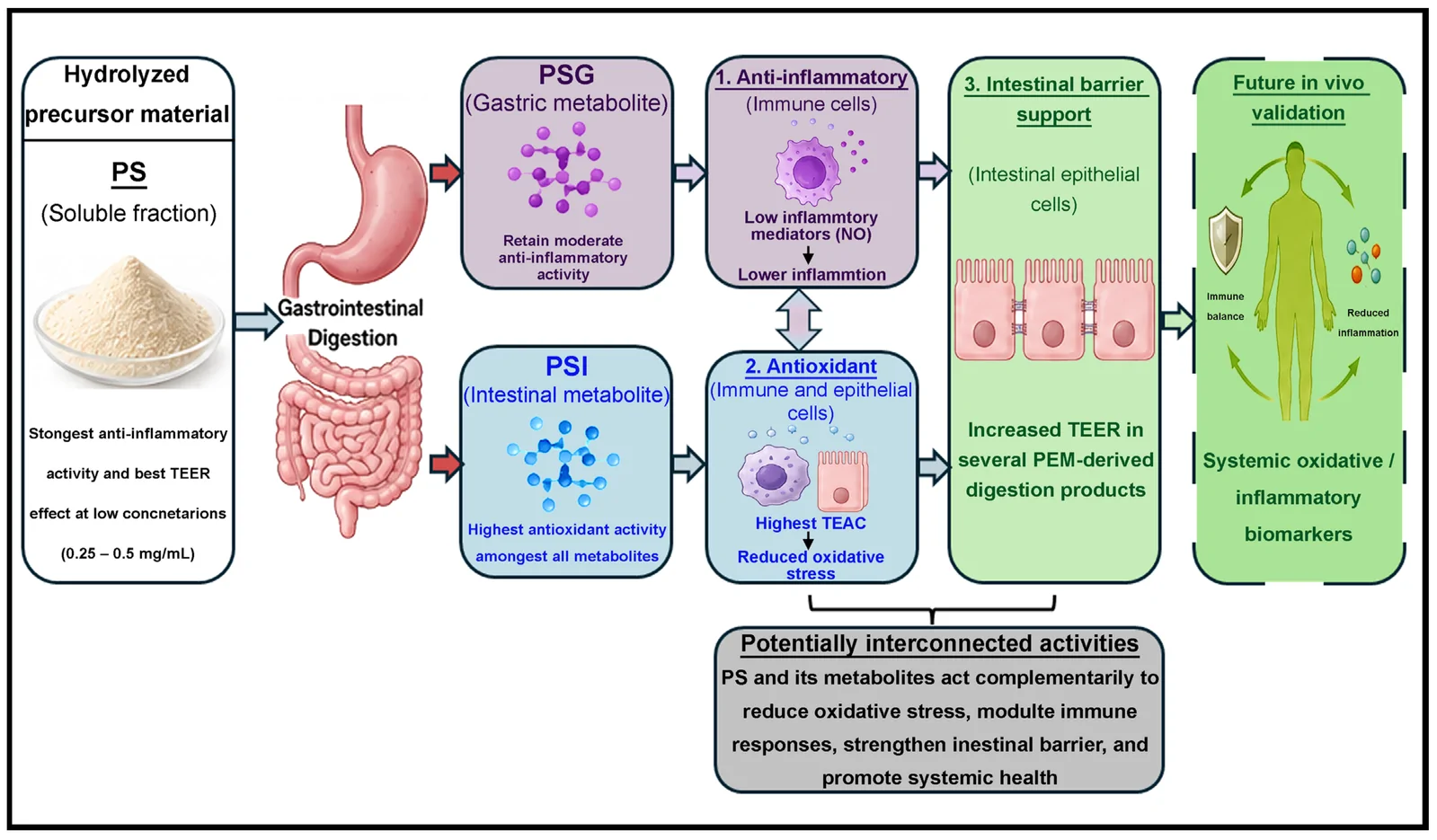
Data-driven conceptual summary of the experimentally observed activities of PEM-derived digestion products. PS exhibited the greatest suppression of LPS-induced NO production, PSI exhibited the highest total antioxidant capacity, and several PEM-derived digestion products increased TEER in differentiated Caco-2 monolayers. The dashed green box indicates potential areas for future in vivo validation rather than conclusions established by the present study.

The soluble fraction of PEM (PS) represents a particularly promising candidate for development as a functional-food ingredient. PS exhibited the strongest suppression of LPS-induced NO production, with the lowest relative NO production observed at 0.5 mg/mL, while also producing the greatest improvement in Caco-2 barrier integrity at the lower concentrations tested, particularly 0.25 and 0.5 mg/mL. Following gastrointestinal digestion, its digestion products PSG and PSI retained anti-inflammatory and barrier-supporting activities, while PSI exhibited the highest antioxidant capacity among all tested digestion products. Although these activities may be mechanistically interconnected through the modulation of oxidative stress and inflammatory responses, the present data do not establish a direct correlation or causal relationship among them (Figure 6).

### 4.6. Limitations and Future Perspectives

Limitations of the present study. The present study has several limitations. First, although the TEAC assay provided a consistent measure for comparing the antioxidant capacity of the different PEM-derived fractions and their simulated gastrointestinal digestion products, the use of a single antioxidant assay does not capture the full range of antioxidant mechanisms. Furthermore, the 3-kDa molecular-weight-cutoff ultrafiltration step used for desalting and concentration leads to the loss of very-low-molecular-weight peptides/digestion products. Therefore, the recovered fractions should be considered protein- and peptide-containing fractions, with SDS-PAGE indicating peptide/protein species predominantly within the ~3.5–50 kDa range. Second, the RAW264.7 macrophage and Caco-2 models provide critical insights into anti-inflammatory and intestinal barrier activity; however, these well-established in vitro systems cannot fully recapitulate the complex interaction between the gastrointestinal environment, immune system, intestinal microbiota, and host metabolism. Third, the observed increases in Caco-2 TEER should consequently be interpreted as evidence of barrier-supportive potential rather than definitive evidence of tight-junction remodeling or altered intestinal permeability in vivo. Finally, the present study also did not identify the specific protein or peptide species responsible for the observed activities or establish causal relationships among antioxidant capacity, suppression of LPS-induced NO production, and TEER responses.

Future research and biological validation. Future research should combine complementary antioxidant assays with peptide profiling, proteomics, fractionation, and sequence identification to characterize candidate bioactive peptides and investigate structure–activity relationships. In addition, the effects of PEM-derived peptides on specific tight-junction proteins, including claudins, occludin, and zonula occludens-1 (ZO-1), along with direct permeability measurements in complementary intestinal epithelial models, should be assessed. Furthermore, three-dimensional (3D) intestinal organoids, co-culture systems incorporating epithelial and immune cells, and microbiota-interacting models could provide more physiologically relevant data. Moreover, in vivo studies are crucial to evaluate bioavailability, efficacy, gastrointestinal stability, safety, and biodistribution, along with establishing whether the exhibited antioxidant, anti-inflammatory, and barrier-supportive activities translate into improved intestinal homeostasis. Most importantly, gut–immune–microbiome interaction, including changes in microbial composition, short-chain fatty acids (SCFAs), inflammatory mediators, oxidative stress biomarkers, and intestinal permeability, should be investigated.

Scale-up and commercialization considerations. Translation into functional food or nutraceutical applications will require standardized processing, reproducible material quality, and consistent functional properties. In this context, a separate patent application related to an upstream process for producing intact ESM feedstock with low residual calcium and controlled microbiological quality has been filed by our laboratory (World Intellectual Property Organization (WIPO) Application No. PCT/CA2024/050768; USPTO Patent Application Serial No. 63/507,329) [24]. This technology is distinct from the PEM hydrolysis strategy investigated in the present study and is intended to provide a controlled and scalable approach for ESM extraction suitable for downstream processing. Such technology was developed to address challenges associated with material processing and reproducibility and may contribute to improved consistency in material quality, purity, and physicochemical characteristics, although further validation is required [24]. Scale-up studies are also needed to establish reproducible hydrolysis conditions, maximize peptide recovery, minimize processing-related degradation, and determine energy requirements along with overall process sustainability. Future development should therefore consider not only the biological efficacy but also industrial scalability, including process cost-effectiveness, microbiological quality, chemical safety, regulatory requirements, consumer acceptability, and life-cycle sustainability.

## 5. Conclusions

The key merit of the current study is the integrated evaluation of ESM size reduction, protein solubilization, fractionation, simulated gastrointestinal digestion, and antioxidant capacity, along with inflammatory and intestinal barrier-associated responses. The study reports that optimized processing of particalized eggshell membrane (PEM), an underexploited eggshell-processing by-product, can generate new soluble peptide-containing fractions with distinct antioxidant capacity and effects on LPS-induced NO production and Caco-2 monolayer TEER. KOH-mediated alkaline hydrolysis achieved approximately 78% protein solubilization and substantially altered the molecular-weight profile of the PEM material, while simulated gastrointestinal digestion further modified the composition and bioactivity of the resulting fractions. Among the assessed fractions, the soluble PEM fraction (PS) exhibited the strongest reduction in LPS-induced NO production, whereas its intestinal digestion product (PSI) demonstrated the highest total antioxidant capacity, reaching more than 65-fold higher as compared to unprocessed PEM. In addition, PS and other PEM-derived fractions and digestion products enhanced the transepithelial electrical resistance (TEER) at selected concentrations in differentiated Caco-2 monolayers without detectable cytotoxicity under the tested conditions, suggesting potential effects on epithelial barrier function.

The demonstrated in vitro bioactivities highlight the potential of PEM-derived ingredients for functional foods and nutraceutical applications targeting oxidative stress and gut health. However, any mechanistic relationship among these activities remains speculative and requires validation in physiologically relevant models and in vivo studies. In general, these findings extend previous Jet-O-Mized ESM utilization by revealing fraction- and digestion-dependent bioactivity. Most importantly, the use of a size-selected PEM feedstock and an alkaline processing strategy provides a defined platform for future process optimization and scale-up. Finally, further characterization of bioactive peptide sequences, gastrointestinal stability, bioavailability, molecular mechanisms, microbiome interactions, safety, scalability, and regulatory requirements will be essential to advance these ESM-derived ingredients toward translational and commercial applications within a circular bioeconomy framework.

## Figures and Tables

**Table 1:** Total antioxidant capacity and anti-inflammatory activity of PEM-derived digestion products following simulated gastrointestinal digestion.

Bioactivities	PEM-Derived Hydrolysate							Control
	Whole Fraction (I)		Soluble Fraction (II)			Pellet Fraction (III)		
	PWG	PWI	PS	PSG	PSI	PPG	PPI	
Total antioxidant capacity (mM TEAC)	1.4 ± 0.0	2.0 ± 0.2	2.5 ± 0.2	3.8 ± 0.2	13.1 ± 2.1	1.5 ± 0.2	2.7 ± 0.5	0.2 ± 0.0 ^#^
Relative NO production (%)	54 ± 8	50 ± 2	47 ± 12	62 ± 8	61 ± 4	75 ± 9	73 ± 11	101 ± 13 ^‡^

TEAC: Trolox equivalent antioxidant capacity; NO: nitric oxide; ^#^: unprocessed particalized eggshell membrane (PEM); ^‡^: lipopolysaccharide (LPS)-treated cells (LC).

**Table 2:** Changes in transepithelial electrical resistance (ΔTEER) between baseline and 24 h in differentiated Caco-2 cell monolayers treated with PEM-derived fractions at different concentrations.

Protein Conc. (mg/mL)	ΔTEER of PEM-Derived Fractions (Ω·cm^2^)							ΔTEER of CTRL (Ω·cm^2^)	
	Whole Fraction (I)		Soluble Fraction (II)			Pellet Fraction (III)		NC	IC
	PWG	PWI	PS	PSG	PSI	PPG	PPI		
0.125	37 ± 7 ^ns^	48 ± 10 ^ns^	28 ± 10 ^ns^	29 ± 5 ^ns^	54 ± 6 *	23 ± 9 ^ns^	33 ± 10 ^ns^	28 ± 10	−192 ± 16
0.25	42 ± 7 ^ns^	49 ± 11 ^ns^	65 ± 12 *	27 ± 3 ^ns^	57 ± 5 *	22 ± 8 ^ns^	34 ± 11 ^ns^		
0.5	43 ± 13 ^ns^	69 ± 9 **	65 ± 9 *	38 ± 2 ^ns^	49 ± 12 ^ns^	26 ± 5 ^ns^	36 ± 15 ^ns^		
1.0	89 ± 10 ****	67 ± 15 **	50 ± 16 ^ns^	60 ± 7 **	-	-	47 ± 16 ^ns^		

ΔTEER: Change in Trans-epithelial electrical resistance, calculated as TEER at 24 h minus baseline TEER (0 h), expressed in Ω·cm^2^ (ohms per square centimetre); positive values indicate increased resistance, whereas negative values indicate decreased resistance. NC: non-treated control; IC: ionomycin-disrupted control; CTRL: control cells. ΔTEER was used to assess the integrity of the Caco-2 intestinal epithelial barrier following exposure to PEM-derived fractions and digestion products. Statistical significance is indicated as *p* < 0.05 (*), *p* < 0.01 (**), and *p* < 0.0001 (****), with ^ns^ indicating not significant. Statistical comparisons were performed between each treatment group and the non-treated control (NC).

## Data Availability

The original contributions presented in this study are included in the article/Supplementary Material. Further inquiries can be directed to the corresponding author.

## Supplementary Materials

The following supporting information can be downloaded at: https://www.mdpi.com/article/10.3390/foods15213906/s1: Table S1: Composition and experimental conditions for simulated gastrointestinal digestion of PEM-derived samples. Simulated gastric digestion (SGD) and simulated intestinal digestion (SID) were performed sequentially using a protocol adapted from Minekus et al. (2014) [26].; Table S2: Summary of eggshell membrane (ESM) processing strategies and reported bioactivities of ESM-derived proteins and peptides; Figure S1: Effect of different acids and concentrations on PEM hydrolysis and solubilization under static conditions; Figure S2: Comparison of time-dependent PEM solubilization under NaOH- and KOH-mediated alkaline conditions at low and high temperatures using 1X, 3X, and 12.5X base concentrations.

## Abbreviations

The following abbreviations are used in this manuscript:

Abbreviation	Full term
ABTS	2,2′-Azino-bis(3-ethylbenzothiazoline-6-sulfonic acid)
ACE	Angiotensin-converting enzyme
ANOVA	Analysis of variance
AOM	Azoxymethane
ATCC	American Type Culture Collection
BCA	Bicinchoninic acid
BBF	Burnbrae Farms
Caco-2	Human colorectal adenocarcinoma cell line
*CLDN1*	Claudin-1
CTRL	Control
DMEM	Dulbecco’s Modified Eagle Medium
DNA	Deoxyribonucleic acid
DPPH	2,2-Diphenyl-1-picrylhydrazyl
DSS	Dextran sodium sulfate
EDTA	Ethylenediaminetetraacetic acid
ES	Eggshell
ESM	Eggshell membrane
ESMH	Eggshell membrane hydrolysate
GIT	Gastrointestinal tract
HCl	Hydrochloric acid
HPEM	Hydrolyzed particalized eggshell membrane
H_2_O_2_	Hydrogen peroxide
IC	Ionomycin control
ICAM-1	Intercellular adhesion molecule-1
IFN-γ	Interferon-gamma
IL	Interleukin
iNOS	Inducible nitric oxide synthase
JEM	Jet-milled eggshell membrane
KOH	Potassium hydroxide
LC	Lipopolysaccharide-treated cell control
LPS	Lipopolysaccharide
M	Molecular-weight marker
MDA	Malondialdehyde
MMP-3	Matrix metalloproteinase-3
Na_2_SO_3_	Sodium sulfite
NaOH	Sodium hydroxide
NF-κB	Nuclear factor kappa B
NEM^®^	Natural Eggshell Membrane
NC	Negative control
NO	Nitric oxide
OD_600_	Optical density at 600 nm
*Ocln*	Occludin
PEM	Particalized eggshell membrane
PPI	Pellet fraction of hydrolyzed PEM following simulated intestinal digestion
PPG	Pellet fraction of hydrolyzed PEM following simulated gastric digestion
PP	Pellet fraction of hydrolyzed PEM
PS	Soluble PEM fraction
PSG	Soluble PEM fraction following simulated gastric digestion
PSI	Soluble PEM following simulated intestinal digestion
PW	Whole PEM hydrolysate fraction
PWI	Whole PEM hydrolysate fraction following simulated intestinal digestion
PWG	Whole PEM hydrolysate fraction following simulated gastric digestion
RAW	264.7 Murine macrophage cell line
ROS	Reactive oxygen species
SCFAs	Short-chain fatty acids
SDS-PAGE	Sodium dodecyl sulfate–polyacrylamide gel electrophoresis
SEP	Soluble eggshell membrane protein
SGD	Simulated gastric digestion
SGF	Simulated gastric fluid
SGID	Simulated gastrointestinal digestion
SID	Simulated intestinal digestion
SIF	Simulated intestinal fluid
SP	Soluble fraction of hydrolyzed PEM
TA	Tannic acid
TBARS	Thiobarbituric acid-reactive substance
TEAC	Trolox equivalent antioxidant capacity
TEER	Transepithelial electrical resistance
*TJP1*	Tight junction protein 1
TNF-α	Tumor necrosis factor-alpha
UPEM	Unprocessed particalized eggshell membrane
WIPO	World Intellectual Property Organization
ZO-1	Zonula occludens-1

## References

[1] Rahman S.Z., Konok F.A., Maliyet C.F., Mia M.F., Rahman T., Rahman A. (2026). Oxidative stress in chronic disease: Mechanisms, therapeutic strategies, and future directions. Adv. Redox Res..

[2] Forman H.J., Zhang H. (2021). Targeting oxidative stress in disease: Promise and limitations of antioxidant therapy. Nat. Rev. Drug Discov..

[3] Sies H., Belousov V.V., Chandel N.S., Davies M.J., Jones D.P., Mann G.E., Murphy M.P., Yamamoto M., Winterbourn C. (2022). Defining roles of specific reactive oxygen species (ROS) in cell biology and physiology. Nat. Rev. Mol. Cell Biol..

[4] Cheung E.C., Vousden K.H. (2022). The role of ROS in tumour development and progression. Nat. Rev. Cancer.

[5] Gao Q., Jiang L., Sun Y., An X., Sun W., Tang S., Kang X., Zhao X., Li Z., Liu C. (2025). Oxidative stress: From molecular studies to clinical intervention strategies. Front. Mol. Biosci..

[6] Sun Y., Wang X., Li L., Zhong C., Zhang Y., Yang X., Li M., Yang C. (2024). The Role of Gut Microbiota in Intestinal Disease: From an Oxidative Stress Perspective. Front. Microbiol..

[7] Kunst C., Schmid S., Michalski M., Tümen D., Buttenschön J., Müller M., Gülow K. (2023). The Influence of Gut Microbiota on Oxidative Stress and the Immune System. Biomedicines.

[8] Chemat F., Abert Vian M., Fabiano-Tixier A.S., Nutrizio M., Režek Jambrak A., Munekata P.E.S., Lorenzo J.M., Barba F.J., Binello A., Cravotto G. (2020). A review of sustainable and intensified techniques for extraction of food and natural products. Green Chem..

[9] Vilas-Boas A.A., Pintado M., Oliveira A.L.S. (2021). Natural Bioactive Compounds from Food Waste: Toxicity and Safety Concerns. Foods.

[10] Granato D., Zabetakis I., Koidis A. (2023). Sustainability, nutrition, and scientific advances of functional foods under the new EU and global legislation initiatives. J. Funct. Foods.

[11] Ahmed T.A.E., Kulshreshtha G., Hincke M.T., Wu J. (2019). Value-Added Uses of Eggshell and Eggshell Membranes. Eggs as Functional Foods and Nutraceuticals for Human Health.

[12] Ahmed T.A.E., Kulshreshtha G., Younes M., Hincke M.T., Mine Y., Guyonnet V., Hatta H., Nau F., Qui N. (2023). Part II. Chemistry and Characterization of Egg Components—Eggshell and Eggshell Membranes. Handbook of Egg Science and Technology.

[13] Hincke M., Ahmed T., Sah M.K. (2026). Eggshell for Biomedical Applications.

[14] Mohseni Ghalehghazi A., Zhong W. (2025). From Nutrition to Innovation: Biomedical Applications of Egg Components. Molecules.

[15] Zhang Y., Wen Z., Wei T., Wu N., Yao Y., Zhao Y., Tu Y. (2026). Eggshell Membrane: Composition, Extraction, Functional Properties, and High-Value Applications in Functional Foods and Nutraceuticals. Compr. Rev. Food Sci. Food Saf..

[16] Ganesan S., Subramani S., Manoj D., Elancheziyan M., Manikandan V., Ramakrishnan S.G., Soundrya S.L., Singh S., Varshney D., Lo H.-M. (2025). A review on eggshell membrane: Structure, functionalization, and its emerging role in bioproduct separation and biorefinery applications. Biomass Bioenergy.

[17] Li Z., Zhang Y., Jin H., Wang S., Chen H., Shu D., Ahn D.U., Huang X. (2024). Application of Steam Explosion for Preparing High Value-Added Antioxidants from Eggshell Membrane Waste. Innov. Food Sci. Emerg. Technol..

[18] Kulshreshtha G., Ahmed T.A.E., Wu L., Diep T., Hincke M.T. (2020). A novel eco-friendly green approach to produce particalized eggshell membrane (PEM) for skin health applications. Biomater. Sci..

[19] Shi Y., Kovacs-Nolan J., Jiang B., Mine Y. (2014). Antioxidant Activity of Enzymatic Hydrolysates from Eggshell Membrane Proteins and Its Protective Capacity in Human Intestinal Epithelial Caco-2 Cells. J. Funct. Foods.

[20] Gu L., Zhang Y., Li J., Chang C., Yang Y., Su Y. (2022). Analysis on Antioxidant Activity and Composition of Eggshell Membrane Peptides Prepared by Extrusion and Enzymatic Hydrolysis. Sci. Technol. Food Ind..

[21] Zhao Q.C., Zhao J.Y., Ahn D.U., Jin Y.G., Huang X. (2019). Separation and Identification of Highly Efficient Antioxidant Peptides from Eggshell Membrane. Antioxidants.

[22] Yoo J., Park K., Yoo Y., Kim J., Yang H., Shin Y. (2014). Effects of Egg Shell Membrane Hydrolysates on Anti-Inflammatory, Anti-Wrinkle, Anti-Microbial Activity and Moisture-Protection. Korean J. Food Sci. Anim. Resour..

[23] Younes M., Ahmed T.A.E., Hammami R., Hincke M.T. (2026). Scalable Jet-O-Mized Eggshell Membrane Processing for Bioactives Promoting Gut Health. Molecules.

[24] Kelly V.R.P., Lanteigne E., Hincke M.T., Hincke M.T., Ahmed T.A.E., Sah M.K. (2026). Recent patents and commercial products reflecting the biomedical applications of eggshell membrane. Eggshell for Biomedical Applications.

[25] Younes M. (2025). Jet-O-Mized Eggshell Membrane Powder: A Novel Hydrolysis Strategy to Unlock Gut-Promoting Bioactivities. Master’s Thesis.

[26] Minekus M., Alminger M., Alvito P., Ballance S., Bohn T., Bourlieu C., Carrière F., Boutrou R., Corredig M., Dupont D. (2014). A standardised static in vitro digestion method suitable for food—An international consensus. Food Funct..

[27] Ahmed T.A.E., Suso H.-P., Hincke M.T. (2017). In-depth comparative analysis of the chicken eggshell membrane proteome. J. Proteom..

[28] Apak R., Güçlü K., Özyürek M., Karademir S.E. (2004). Novel Total Antioxidant Capacity Index for Dietary Polyphenols and Vitamins C and E, Using Their Cupric Ion Reducing Capability in the Presence of Neocuproine: CUPRAC Method. J. Agric. Food Chem..

[29] Valdez J.C., Cho J., Bolling B.W. (2020). Aronia berry inhibits disruption of Caco-2 intestinal barrier function. Arch. Biochem. Biophys..

[30] Mottawea W., Yousuf B., Sultan S., Ahmed T., Yeo J., Hüttmann N., Li Y., Bouhlel N.E., Hassan H., Zhang X. (2025). Multi-level analysis of gut microbiome extracellular vesicles-host interaction reveals a connection to gut-brain axis signaling. Microbiol. Spectr..

[31] Zhou Y.H., Ma M.H., Cai Z.X., Li T. (2009). Spectral Analysis of Soluble Egg Shell Membrane Protein and Antioxidant Activities of Its Enzymatic Hydrolysates. Food Sci..

[32] Huang X., Zhou Y., Ma M., Cai Z., Li T. (2010). Chemiluminescence Evaluation of Antioxidant Activity and Prevention of DNA Damage Effect of Peptides Isolated from Soluble Eggshell Membrane Protein Hydrolysate. J. Agric. Food Chem..

[33] Zhu L., Xiong H., Huang X., Guyonnet V., Ma M., Chen X., Zheng Y., Wang L., Hu G. (2022). Identification and Molecular Mechanisms of Novel Antioxidant Peptides from Two Sources of Eggshell Membrane Hydrolysates Showing Cytoprotection against Oxidative Stress: A Combined In Silico and In Vitro Study. Food Res. Int..

[34] Wang S., Abou-Elsoud M., Li Z., Chen J., Ren S., Shu D., Liu M., Huang X. (2025). Structural Modulation of Eggshell Membrane Hydrolysates by Tannic Acid: Simultaneous Enhancement of Antioxidant and Emulsifying Properties. Food Chem..

[35] Cai K., Hou B., Hu Y., He X., Shan Y. (2025). Subcritical Water Hydrolysis of Eggshell Membrane and Its Physicochemical Characteristics. Food Chem..

[36] Kim J.W., Lee D.H., Lee K.W., Na I.S., Lee N.Y., Kim J.K., Chun Y.S., Yoon N.K., Kim B.K., Yang S.K. (2024). Profiling Bioactive Components of Natural Eggshell Membrane (NEM) for Cartilage Protection and Its Protective Effect on Oxidative Stress in Human Chondrocytes. Int. J. Mol. Sci..

[37] Jain S., Anal A.K. (2017). Production and Characterization of Functional Properties of Protein Hydrolysates from Egg Shell Membranes by Lactic Acid Bacteria Fermentation. J. Food Sci. Technol..

[38] Bayraktar O., Galanakis C.M., Aldawoud T.M.S., Ibrahim S.A., Köse M.D., Uslu M.E. (2021). Utilization of Eggshell Membrane and Olive Leaf Extract for the Preparation of Functional Materials. Foods.

[39] Benson K.F., Ruff K.J., Jensen G.S. (2012). Effects of Natural Eggshell Membrane (NEM) on Cytokine Production in Cultures of Peripheral Blood Mononuclear Cells: Increased Suppression of Tumor Necrosis Factor-α Levels after In Vitro Digestion. J. Med. Food.

[40] Ruff K.J., Durham P.L., O’Reilly A., Long F.D. (2015). Eggshell Membrane Hydrolyzates Activate NF-κB In Vitro: Possible Implications for In Vivo Efficacy. J. Inflamm. Res..

[41] Shi Y., Rupa P., Jiang B., Mine Y. (2014). Hydrolysate from Eggshell Membrane Ameliorates Intestinal Inflammation in Mice. Int. J. Mol. Sci..

[42] Jia H., Hanate M., Aw W., Itoh H., Saito K., Kobayashi S., Hachimura S., Fukuda S., Tomita M., Hasebe Y. (2017). Eggshell Membrane Powder Ameliorates Intestinal Inflammation by Facilitating the Restitution of Epithelial Injury and Alleviating Microbial Dysbiosis. Sci. Rep..

[43] Vuong T.T., Rønning S.B., Suso H.P., Schmidt R., Prydz K., Lundström M., Moen A., Pedersen M.E. (2017). The Extracellular Matrix of Eggshell Displays Anti-Inflammatory Activities through NF-κB in LPS-Triggered Human Immune Cells. J. Inflamm. Res..

[44] Yu M., Park C., Son Y.B., Jo S.E., Jeon S.H., Kim Y.J., Han S.B., Hong J.T., Son D.J. (2024). Time-Dependent Effect of Eggshell Membrane on Monosodium-Iodoacetate-Induced Osteoarthritis: Early-Stage Inflammation Control and Late-Stage Cartilage Protection. Nutrients.

[45] Mao X., Xu L., Cao Y., Wang M., Wang W. (2026). Eggshell Membrane Peptides Alleviate IL-1β-Induced Inflammatory Responses and Extracellular Matrix Degradation in Canine Chondrocytes by Inhibiting the NF-κB Signaling Pathway. Animals.

[46] Moran J. (2026). Anti-Inflammatory Effects of Soluble Eggshell Membrane Powder on LPS-Stimulated Caco-2 Cells: Modulation of Proinflammatory Cytokine Gene Expression. Am. J. Clin. Exp. Med..

[47] Rønning S.B., Carlsen H., Rocha S.D.C., Rud I., Solberg N., Høst V., Veiseth-Kent E., Arnesen H., Bergum S., Kirkhus B. (2024). Dietary Intake of Micronized Avian Eggshell Membrane in Aged Mice Reduces Circulating Inflammatory Markers, Increases Microbiota Diversity, and Attenuates Skeletal Muscle Aging. Front. Nutr..

[48] Jia H., Lyu W., Hirota K., Saito E., Miyoshi M., Hohjoh H., Furukawa K., Saito K., Haritani M., Taguchi A. (2022). Eggshell Membrane Modulates Gut Microbiota to Prevent Murine Pre-Cachexia through Suppression of T Helper Cell Differentiation. J. Cachexia Sarcopenia Muscle.

[49] Yang Y., Jia H., Lyu W., Furukawa K., Li X., Hasebe Y., Kato H. (2022). Dietary Eggshell Membrane Powder Improves Survival Rate and Ameliorates Gut Dysbiosis in Interleukin-10 Knockout Mice. Front. Nutr..

[50] Yin W., Ma S., Igarashi M., Li X., Suzuki-Kemuyama N., Hasebe Y., Kato H., Jia H. (2026). Dietary Eggshell Membrane Modulates Gut Microbiota and Alleviates AOM/DSS-Induced Colonic Inflammation in Mice. Biosci. Rep..

[51] Martins-Gomes C., Nunes F.M., Silva A.M. (2024). Natural Products as Dietary Agents for the Prevention and Mitigation of Oxidative Damage and Inflammation in the Intestinal Barrier. Antioxidants.

[52] Esworthy R.S. (2025). Enzymatic Oxidants, Antioxidants, and Inflammatory Bowel Disease. Appl. Biosci..

[53] Neurath M.F., Artis D., Becker C. (2025). The intestinal barrier: A pivotal role in health, inflammation, and cancer. Lancet Gastroenterol. Hepatol..

[54] Barakat H., Sakr S.S., Alfheeaid H.A., Alsaleem K.A., Alhomaid R.M., El-Messery T.M., Ebeid T.A., Abdul-Hafeez E.Y. (2026). Oxidative Stress–Gut Microbiome Crosstalk: Intestinal Redox Imbalance and Probiotics Therapeutic Potential. Antioxidants.

[55] Yang-Jensen S.K., Nägele N.S., Jensen B.A.H. (2025). From gut to blood: Barrier dysfunction as a driver of systemic low-grade inflammation in cardiometabolic disease. Am. J. Physiol. Cell Physiol..

