# Supplementary material for: Antioxidant Bioactivity of Particalized Eggshell Membrane (PEM) Targeting Potential Functional Food Applications
Source: Foods. 2026 Oct 30;15(21):3906. doi: 10.3390/foods15213906 (PMC13763434; doi:10.3390/foods15213906)
Supplement: Supplementary file 1 [file foods-15-03906-s001.zip › foods-4603800-supplementary.pdf]

foods

MDPI

Supplementary Materials_R1

Antioxidant Bioactivity of Particalized Eggshell Membrane (PEM) Targeting Potential Functional Food Applications

Tamer A. E. Ahmed[1,†], Manar Younes[1,†], Riadh Hammami[2], and Maxwell T. Hincke[1,3,*]

[1] Department of Cellular and Molecular Medicine, Faculty of Medicine, University of Ottawa, Ottawa, ON K1H 8M5, Canada; tahmed@uottawa.ca (T.A.E.A.); myoun141@uottawa.ca (M.Y.)
[2] School of Nutrition Sciences, Faculty of Health Sciences, University of Ottawa, Ottawa, ON K1H 8M5, Canada; riadh.hammami@uottawa.ca (R.H.)
[3] Department of Innovation in Medical Education, Faculty of Medicine, University of Ottawa, Ottawa, ON K1H 8M5, Canada
* Correspondence: mhincke@uottawa.ca; Tel. : +1-613-562-5800 (ext. 8193)
† Equal contribution.

Table S1. Composition and experimental conditions for simulated gastrointestinal digestion of PEM-derived samples. Simulated gastric digestion (SGD) and simulated intestinal digestion (SID) were performed sequentially using a protocol adapted from Minekus et al. (2014).

**Component**	**Gastric digestion (SGD)**	**Intestinal digestion (SID)**
**Starting material volume**	10 mL of PEM suspension	20 mL PEM-containing gastric chyme
**Simulated digestive fluid added volume**	7.5 mL Simulated gastric fluid (SGF)	11 mL Simulated intestinal fluid (SIF)
**Enzyme**	Pepsin: 2,000 U/mL	**Pancreatin:** 100 U/mL
**Bile**	—	10 mM
**$CaCl_2$**	75 μM	300 μM
**pH adjustment**	HCl	NaOH
**pH (Final)**	3.0	7.0
**Temperature**	37 °C	37 °C
**Time**	2 h	4 h

The reported enzyme activities, bile concentrations, and $CaCl_2$ concentrations refer to final digestion-mixture concentrations.

Table S2. Summary of ESM processing strategies and reported bioactivities of ESM-derived proteins and peptides.

**Feedstock ESM**	**Feedstock ESM Nature**	**Chemical/thermal hydrolysis and modification strategies**	**Identified antioxidant activity**	**Other reported activity**	**Ref.**
SEP (3-MPA/ acetic acid, 90 °C)	Soluble	Five different enzymes: alkaline protease, trypsin, neutral protease, papain, and pepsin.	Superoxide radicals scavenging.	None.	[31]
	Soluble	Five different enzymes: Alcalase, trypsin, Neutrase, caroid, and pepsin.	Superoxide radicals &hydroxyl scavenging.	Prevent DNA damage.	[32]
ESM hydrolysate (NaOH / Ethanol, 55 °C)	Soluble	Two enzymes: Alcalase and Protease S.	DPPH, hydroxyl-radical scavenging, reducing power, Fe^{2+} chelation, cellular antioxidant activity.	IL-8 suppression; increased GSH levels in Caco-2 cells.	[19]
ESM	Powder	Five different enzymes: alkaline protease, pepsin, chymotrypsin, neutral protease, papain in the presence of Na_2SO_3.	DPPH, ABTS, reducing power, Fe^{2+} chelation, TAA, TBARS.	None.	[21]
Fresh and hatched ESM	Powder	Enzymatic: Alcalase/55 °C.	ORAC, ABTS, cellular ROS/MDA reduction.	Cytoprotection.	[33]
ESM	Flakes	Steam explosion, enzymatic hydrolysis (alkaline protease)/ 55 °C.	DPPH, total antioxidant capacity, synergistic antioxidant activity with gallic acid (GA).	ROS reduction in L929 cells.	[17]
ESM	Flakes	Steam explosion, enzymatic hydrolysis (Alcalase)/ 55 °C, modification with tannic acid (TA).	Free-radical scavenging; Fe^{2+} chelation.	Reduced lipid oxidation.	[34]
ESM	Powder	Subcritical water extraction, 180–210 °C.	Free-radical-scavenging activity.	None.	[35]
NEM®	Powder	Simulated gastric digestion.	ABTS/TEAC, increased SOD, cellular ROS reduction.	Reduced H_2O_2-induced apoptosis, cartilage protection	[36]
ESM	Powder	*L. plantarum* fermentation.	DPPH scavenging; reducing power.	ACE inhibition; antibacterial activity.	[37]
JEM (Jet milling)	Powder	KOH.	Total antioxidant activity.	Antibacterial, anti-inflammatory, and intestinal transport activities.	[23]
ESM	Intact	Adsorption/loading with olive-leaf polyphenols.	TEAC/overall antioxidant capacity	Functional material development.	[38]

ESM, eggshell membrane; SEP, soluble eggshell membrane protein; 3-MPA, 3-mercaptopropionic acid; ESMH, eggshell membrane hydrolysate; TA, tannic acid; GA, gallic acid; SW, subcritical water; NEM, Natural ESM; JEM, jet-milled eggshell membrane; KOH, potassium hydroxide; NaOH, sodium hydroxide; Na_2SO_3, sodium sulfite; DPPH, 2,2-diphenyl-1-picrylhydrazyl; ABTS, 2,2′-azinobis-(3-ethylbenzothiazoline-6-sulfonic acid); TEAC, Trolox equivalent antioxidant capacity; H_2O_2, hydrogen peroxide; ORAC, oxygen radical absorbance capacity; ROS, reactive oxygen species; MDA, malondialdehyde; TAA, total antioxidant activity; TBARS, thiobarbituric acid reactive substances; GSH, glutathione; SOD, superoxide

https://doi.org/10.3390/foods15213906

dismutase; IL-8, interleukin-8; ACE, angiotensin-converting enzyme; L929, mouse fibroblast cell line; Caco-2, human colorectal adenocarcinoma cell line; RAW264.7, mouse macrophage cell line.

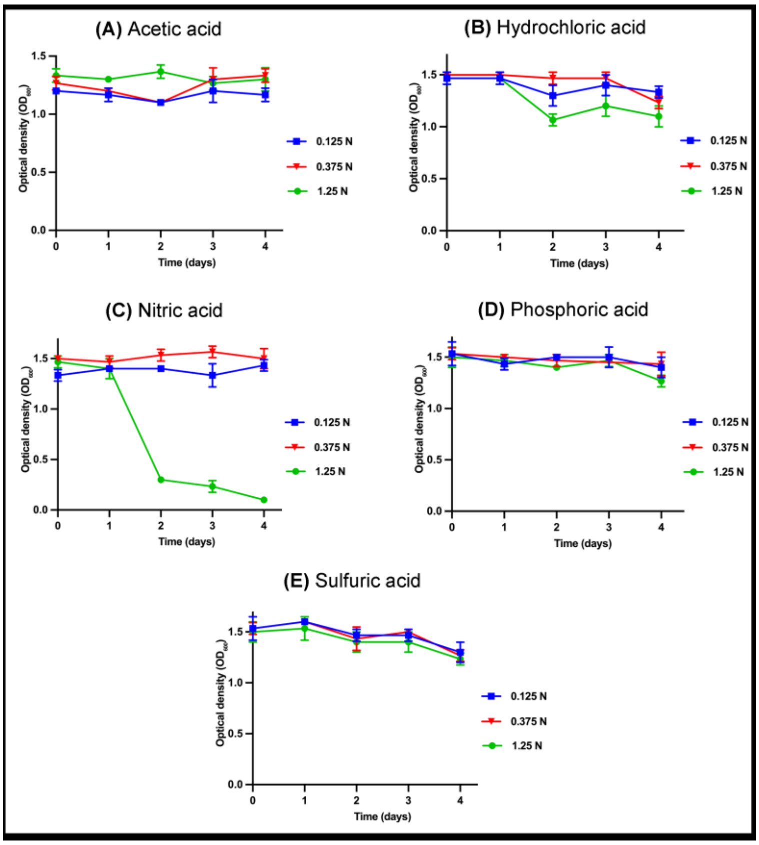

Figure S1. Effect of various acids with different concentrations on PEM hydrolysis and solubilization under static conditions. Time-dependent changes in optical density (OD_{600}), used as an indicator of changes in the solubility of the suspended PEM fraction, during treatment with acetic acid (A), hydrochloric acid (B), nitric acid (C), phosphoric acid (D), and sulfuric acid (E) at 0.125, 0.375, and 1.25 N. Samples were incubated at 50 °C under static conditions without agitation or stirring for up to 4 days at five measurement time points (days 0, 1, 2, 3, and 4). Data are presented as mean ± SD.

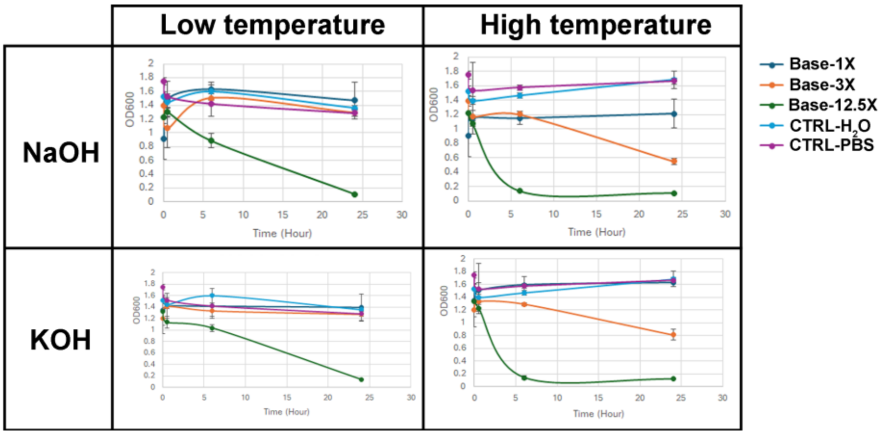

Figure S2. Comparison of time-dependent PEM solubilization under NaOH- and KOH-mediated alkaline conditions at low and high temperatures using 1X, 3X, and 12.5X base concentrations. OD600 was monitored over 24 h as an indicator of changes in turbidity and PEM solubilization. Both NaOH and KOH showed similar overall time-dependent profiles across the tested base concentrations, with the 12.5X condition showing the most pronounced decrease in OD600, particularly at high temperature, whereas 1X and 3X conditions showed comparatively limited changes over time. CTRL-H_2O and CTRL-PBS represent the water and phosphate-buffered saline controls, respectively. Data are presented as

mean ± SD. This experiment was conducted as a preliminary screening of base concentration and temperature conditions and was not intended to establish the superiority of KOH over NaOH.

References

17. Li, Z.; Zhang, Y.; Jin, H.; Wang, S.; Chen, H.; Shu, D.; Ahn, D.U.; Huang, X. Application of Steam Explosion for Preparing High Value-Added Antioxidants from Eggshell Membrane Waste. Innov. Food Sci. Emerg. Technol. 2024, 91, 103552. https://doi.org/10.1016/j.ifset.2023.103552
19. Shi, Y.; Kovacs-Nolan, J.; Jiang, B.; Mine, Y. Antioxidant Activity of Enzymatic Hydrolysates from Eggshell Membrane Proteins and Its Protective Capacity in Human Intestinal Epithelial Caco-2 Cells. J. Funct. Foods 2014, 10, 35–45. https://doi.org/10.1016/j.jff.2014.05.004
21. Zhao, Q.C.; Zhao, J.Y.; Ahn, D.U.; Jin, Y.G.; Huang, X. Separation and Identification of Highly Efficient Antioxidant Peptides from Eggshell Membrane. Antioxidants [M65.1][TA65.2]2019, 8, 495. https://doi.org/10.3390/antiox8100495
23. Younes, M.; Ahmed, T.A.E.; Hammami, R.; Hincke, M.T. Scalable Jet-O-Mized Eggshell Membrane Processing for Bioactives Promoting Gut Health. Molecules 2026, 31, 1217. https://doi.org/10.3390/molecules31071217
31. Zhou, Y.H.; Ma, M.H.; Cai, Z.X.; Li, T. Spectral Analysis of Soluble Egg Shell Membrane Protein and Antioxidant Activities of Its Enzymatic Hydrolysates. Food Sci. 2009, 30, 13–16. https://www.spkx.net.cn/EN/Y2009/V30/I15/13
32. Huang, X.; Zhou, Y.; Ma, M.; Cai, Z.; Li, T. Chemiluminescence Evaluation of Antioxidant Activity and Prevention of DNA Damage Effect of Peptides Isolated from Soluble Eggshell Membrane Protein Hydrolysate. J. Agric. Food Chem. 2010, 58, 12137–12142. https://doi.org/10.1021/jf101728d
33. Zhu, L.; Xiong, H.; Huang, X.; Guyonnet, V.; Ma, M.; Chen, X.; Zheng, Y.; Wang, L.; Hu, G. Identification and Molecular Mechanisms of Novel Antioxidant Peptides from Two Sources of Eggshell Membrane Hydrolysates Showing Cytoprotection against Oxidative Stress: A Combined In Silico and In Vitro Study. Food Res. Int. 2022, 157, 111266. https://doi.org/10.1016/j.foodres.2022.111266
34. Wang, S.; Abou-Elsoud, M.; Li, Z.; Chen, J.; Ren, S.; Shu, D.; Liu, M.; Huang, X. Structural Modulation of Eggshell Membrane Hydrolysates by Tannic Acid: Simultaneous Enhancement of Antioxidant and Emulsifying Properties. Food Chem. 2025, 470, 143827. https://doi.org/10.1016/j.foodchem.2025.143827
35. Cai, K.; Hou, B.; Hu, Y.; He, X.; Shan, Y. Subcritical Water Hydrolysis of Eggshell Membrane and Its Physicochemical Char-acteristics. Food Chem. 2025, 464, 142848. https://doi.org/10.1016/j.foodchem.2025.142848
36. Kim, J.W.; Lee, D.H.; Lee, K.W.; Na, I.S.; Lee, N.Y.; Kim, J.K.; Chun, Y.S.; Yoon, N.K.; Kim, B.K.; Yang, S.K.; et al. Profiling Bioactive Components of Natural Eggshell Membrane (NEM) for Cartilage Protection and Its Protective Effect on Oxidative Stress in Human Chondrocytes. Int. J. Mol. Sci. 2024, 25, 11304. https://doi.org/10.3390/ijms252011304
37. Jain, S.; Anal, A.K. Production and Characterization of Functional Properties of Protein Hydrolysates from Egg Shell Mem-branes by Lactic Acid Bacteria Fermentation. J. Food Sci. Technol. 2017, 54, 1062–1072. https://doi.org/10.1007/s13197-017-2530-y
38. Bayraktar, O.; Galanakis, C.M.; Aldawoud, T.M.S.; Ibrahim, S.A.; Köse, M.D.; Uslu, M.E. Utilization of Eggshell Membrane and Olive Leaf Extract for the Preparation of Functional Materials. Foods 2021, 10, 806. https://doi.org/10.3390/foods10040806
